# An Integrated Theoretical/Experimental Study of Quinolinic–Isoquinolinic Derivatives Acting as Reversible Electrochromes

**DOI:** 10.3390/ma10070802

**Published:** 2017-07-15

**Authors:** Mauro Sassi, Matteo M. Salamone, Luca Beverina, Gianluca Longoni, Claudio Fontanesi, Davide Vanossi, Luigi Cigarini, Riccardo Ruffo

**Affiliations:** 1 Dipartimento di Scienza dei Materiali, Università degli Studi di Milano Bicocca, via Cozzi 55, 20125 Milano, Italy; mauro.sassi@mater.unimib.it (M.S.); matteo.salamone@unimib.it (M.M.S.); luca.beverina@unimib.it (L.B.); g.longoni11@campus.unimib.it (G.L.); 2 Dipartimento di Ingegneria Enzo Ferrari, Università degli Studi di Modena e Reggio Emilia, via Vivarelli 10, 41125 Modena, Italy; claudio.fontanesi@unimore.it (C.F.); davide.vanossi@unimore.it (D.V.); luigi33374@gmail.com (L.C.)

**Keywords:** organic electrochromic materials, violenes, quinoline, isoquinoline

## Abstract

A series of compounds, featuring an ethenylic bridge and quinoline and isoquinoline end capping units possessing systematically varied substitution patterns, were prepared as molecular materials for electrochromic applications. The different structures were optimized in order to maximize the electrochromic contrast in the visible region, mostly by achieving a completely UV-absorbing oxidized state. Density functional theory (DFT) calculations are exploited in order to rationalize the correlation between the molecular structure, the functional groups’ electronic properties, and the electrochemical behavior. It is shown that the molecular planarity (i.e. ring/ring π conjugation) plays a major role in defining the mechanism of the electrochemical charge transfer reaction, while the substituent’s nature has an influence on the LUMO energy. Among the compounds here studied, the (E)-10-methyl-9-(2-(2-methylisoquinolinium-1-yl)-vinyl)-1,2,3,4-tetrahydroacri-dinium trifluoromethanesulfonate derivative shows the most interesting properties as an electrochromophore.

## 1. Introduction

Organic electrochromic materials represent a well-established alternative to inorganic systems, which are currently used in most electrochromic devices. One of their most intriguing properties is the possibility to tailor the hue of their colored states through functionalization, which allows to expand, almost endlessly, the color palette of the existing electrochromes [1]. Applications like smart windows, rearview mirrors, or sunglasses require the material to have a highly transmissive bleached state and a colored one featuring a neutral hue to prevent color distortion in the transmitted light. Conjugated polymers, which have recently received more attention as electrochromic system, are still far from reaching these targets. The best example in the literature shows a transmittance of the colorless state ranging from 45% to 70% for films having a reduced state transmittance in the proper range for their applications [2]. The residual visible absorption in the bleached state is a consequence of the tailing of the polaronic/bipolaronic bands of the oxidized polymers in the Vis-NIR region. This intrinsic limitation of polymeric compounds triggered the parallel development of different approaches. Indeed, organic electrochromic molecular materials offer several examples of molecules possessing a colorless bleached state whose absorption is fully localized in the UV region, along with a colored state accessible via a reduction process [3]. Violenes probably are the best known examples of performing molecular materials, though with a relevant limit: their colored state is a radical. This has consequences in terms of environmental stability and durability [4].

Despite the huge number of different violene-type molecules studied, just a few of them have undergone some kind of development in the direction of a device application: viologens, N,N,N′,N′-tetraphenyl-1,4-phenylenediamine, 2,2′-azo-3,3′-dialkylbis(benzothiazol-3-ium), and phenothiazine. The first class, in particular, is the most studied, with a huge variety of different functionalizations in order to modulate their solubility and color, and it is the only one to find application in solution-based electrochromic devices. Nevertheless, stability remains an issue.

An improved molecular material should fulfill the following requirements: (i) an ability to switch reversibly between a colored and a colorless form; (ii) a closed-shell structure in both forms; (iii) ease of functionalization for further color tuning; (iv) chemical and photochemical stability (at least to visible radiation); and (v) easy synthetic access starting from cheap precursors. In particular, the second requirement is difficult to achieve since most of the violene-type structures show a two-step redox behavior with an Intermediate Radical Form (IRF) having a wide stability range. The overall electrochemical process from the neutral reduced form (Red) to the closed-shell dication one (Ox) can be expressed as:

$$\mathrm{Red}\overset{E_{1}}{↔}\mathrm{IRF}\overset{E_{2}}{↔}\mathrm{Ox}$$

where IRF represents the radical cation [5]. The reaction can be discussed looking at the electrochemical properties of the system, in particular, when the two redox waves are sufficiently spaced apart, the difference in their potentials is a measure of the stability of the IRF [6]:

$$K_{\mathrm{IRF}}=e^{\left(\frac{F\left(E_{2}-E_{1}\right)}{\mathrm{RT}}\right)}=\frac{{\left[\mathrm{IRF}\right]}^{2}}{\left[\mathrm{Red}\right]\left[\mathrm{Ox}\right]}$$

where E_1_ and E_2_ are the half wave potentials of the corresponding electrochemical processes. As an example, N,N′-dimethyl-4,4′-bipyridine has a K_IRF_ = 1.3 × 10^7^ and a peak separation of 0.42 V. Strategies to lower the stability of the radical cation were suggested using the proper molecular design of the target violene structure. Among other approaches to reduce the K_IRF_, two different modifications have been exploited: increasing the conjugation length between end capping units, or benzocondensation [6]. This latter approach is particularly interesting because violenes systems that exhibit bielectronic transitions between the two forms can be obtained by an extension of the viologen structure.

**Figure 1:**
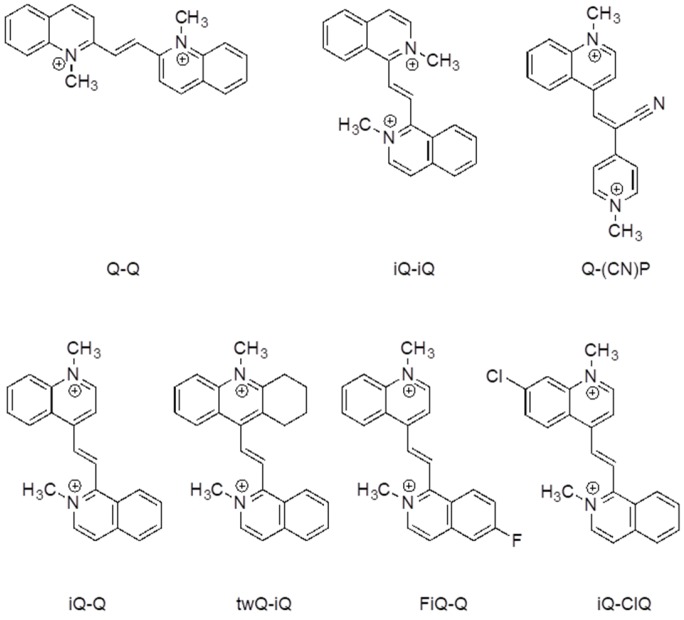
Quinolinium (Q) and isoquinolinium (iQ) substituted ethene derivatives prepared and characterized in this work.

In this contribution, the synthesis of a library of different diazinium ethenes is described, along with the characterization of their electrochemical and spectroelectrochemical properties. The aim of the study is the formulation of structural properties relationships for the achievement of bielectronic behavior along with a completely colorless oxidized form. The azinium derivatives shown in Figure 1 pertain to the class of Weitz-type redox systems. Both end capping units are part of a cyclic π-system that has an aromatic character in the oxidized form. The experimental data are also compared and discussed with ab initio calculation results to correlate the molecular geometry and the electronic structure with the optical and the electrochemical properties. In particular, the effect of the nature of the end groups (heteroaromatic azines), the conjugation pattern (heavily influenced by the substitution patterns of the azines), and the presence of substituent are detailed.

## 2. Results

### 2.1. Molecular Design

**Figure 2:**
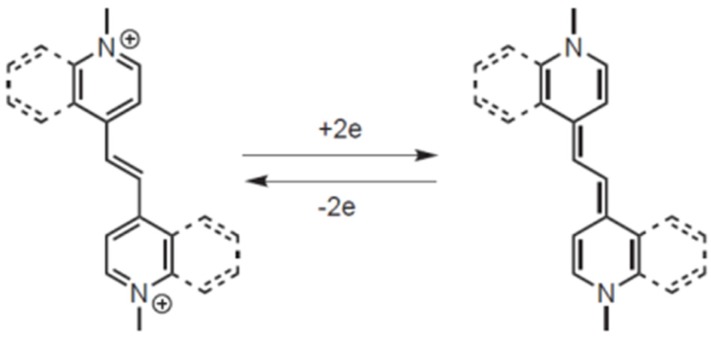
Schematic representation of the stabilizing effect of benzocondensation on the reduced form of diazinium ethene.

In the introduction, it was briefly mentioned that in the series of diazinium ethenes developed by Hünig et al., an effect related to benzocondensation emerges, resulting in a shift of the reduction potentials to less negative values [4,5]. This can be attributed to the strongly π-accepting capabilities of the quinoline ring with respect to pyridine (lower LUMO and lower HOMO), and to the aromatic stabilization of the reduced form. It has to be considered, in fact, that the aromatic-to-quinoid transition takes place upon reduction to the fully reduced form. Benzocondensation allows us to retain a certain grade of aromatic stabilization, as illustrated in Figure 2, according to the fact that one of the two rings in the terminal group gains full benzenoid aromaticity upon reduction.

Despite the huge number of Weitz-type violene systems studied by Hünig et al., for some reason, they always limited their investigation to symmetric systems, even in those cases where asymmetric derivatives were synthetically accessible. We decided to take full advantage of the benzocondensation effect, also exploring the design of asymmetric structures in order to further increase the tailoring of the properties by mixing different heterocycles with similar redox potentials. The effect of the presence of different substituents on the electrochemical and spectroelectrochemical properties was also studied, with the aim of developing the optimal strategy to tune the color of its reduced state. For this purpose, derivates twQ-iQ, FiQ-Q, and iQ-ClQ (Figure 1) were also prepared. In detail, to maximize the effect of substitution, the halogen groups in the derivatives FiQ-Q and iQ-ClQ were placed in the p-position with respect to the position of the ethylene bridge. In the compound twQ-iQ, the choice of the tetrahydroacridine end group used to explore the effect of the alkyl donating groups was dictated by synthetic convenience. The introduction of electron withdrawing groups stimulates a shift of the redox wave to higher potential values, as expected. A relevant feature of azinic derivatives, particularly those obtained through the stepwise approach we employed, is the introduction of different functional solubilizing chains in the nitrogen atoms. In fact, along with solubility, those chains enabled conjugation with other, possibly electro-active, components, as some of us have recently showed.

### 2.2. Electrochemical and Spectroelectrochemical Properties

**Figure 3:**
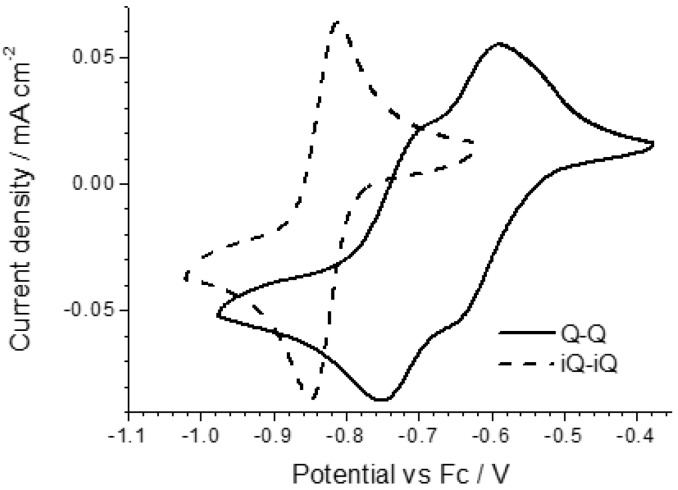
Cyclic voltammogram of the symmetric quinolinium (Q-Q)-based and isoquinolinium (iQ-iQ)-based ethenes.

**Figure 4:**
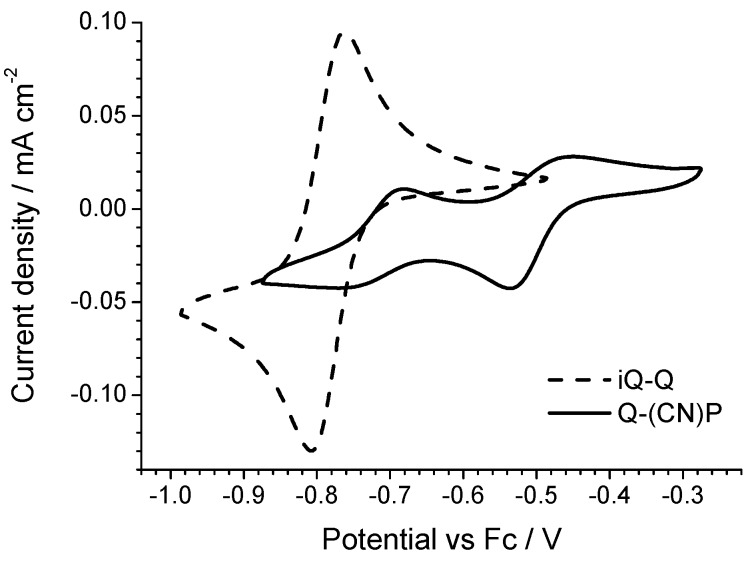
Cyclic voltammograms of the iQ-Q and Q-(CN)P asymmetric molecules.

The electrochemical properties of the diazinium ethene compounds were obtained by cyclic voltammetry measurements. The voltammograms of the symmetric Q-Q and iQ-iQ molecules are depicted in Figure 3. Both systems exhibit reversible redox behavior. In particular, the presence of two distinct redox waves, corresponding to the E_1_ and E_2_ processes (see Introduction), is evident in the Q-Q compound. The process E_1_ is characterized by a oxidation shoulder and a reduction peak, while E_2_ by a reduction shoulder and an oxidation peak. To separate the two processes, we also performed a differential pulsed voltammetry (DPV) analysis (Figure SI_1). The peak to peak separation, consistent for the both reductions and oxidations, is 100 ± 5 mV, which can be used to estimate the K_IRF_ as 50 ± 10. In contrast, a highly reversible two-electron process is involved in iQ-iQ, as testified by the low peak separation observed (ΔE = 38 mV) in the sharply-peaked redox waves. Similar behavior is observed in the asymmetric compound iQ-Q (Figure 4). In this case, the measured peak separation is 40 mV, thus confirming the involvement of a two-electron process. Q-(CN)P, conversely, has a less negative reduction potential, as expected by the presence of the strong electron withdrawing –CN group. However, the electrochemical process shows two partially irreversible mono-electronic waves. Indeed, in each process, the separation between the reduction and oxidation peaks is about 80 mV, larger than the Nernstian theoretical value. However, the voltage difference between the oxidation peaks of E_1_ and E_2_ is consistent with the difference between the reduction peaks, and was calculated as 230 ± 5 mV, which gives a K_IRF_ value of 7.8 × 10^3^ ± 1.7 × 10^3^, two orders of magnitude larger than for the Q-Q compound. From the reduction potential waves, it is also possible to calculate the corresponding LUMO energy levels of the dication forms, assuming the vacuum level to coincide with the ferrocene potential in the electrolyte is (−5.2 eV).

The electrochemical data are summarized in Table 1, wherein E_1_ and E_2_ correspond to the first and second redox processes, according to the above reported equation (see Introduction). Absolute redox potentials, and thus the corresponding LUMO levels, can be relatively straightforwardly rationalized in terms of molecular structure. In the case of the symmetric structures, indeed, the larger electron withdrawing character of the quinolinic rings leads to a higher reduction potential and a lower LUMO level. Dealing with the asymmetric structures, the LUMO level of the iQ-Q compound is intermediate between those of the corresponding symmetric systems (iQ-iQ and Q-Q). As for Q-(CN)P, the strong electron withdrawing character of the cyanopyridinic substituent results in the highest reduction potential and thus the lowest LUMO level.

It is more difficult to rationalize in such simple terms why some derivatives feature mono- instead of two-electron redox processes. The computational investigation reported below was specifically targeted to investigate this issue.

**Figure 5:**
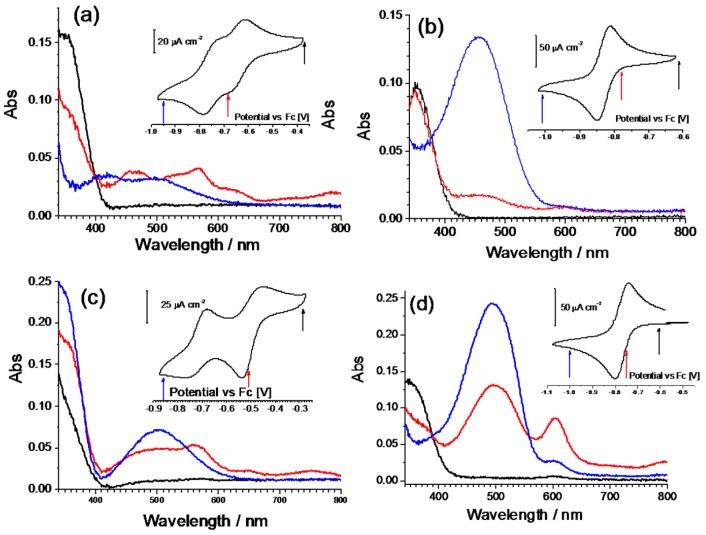
Spectroelectrochemistry of the Q-Q (**a**), iQ-iQ (**b**), Q-(CN)P (**c**) and iQ-Q (**d**) compounds. Spectra were measured at fixed potential corresponding to the points marked in the coefficient of variation (CV) curves (onset). The sequence is: oxidized (black), intermediate (red), and reduced (blue) state.

These compounds were further characterized by an in situ spectroelectrochemical measurement using a 10^–4^ M solution in a thin layer cell. All the dication forms give a strong UV absorption centered at about 350 nm (black lines in Figure 5), only the Q-(CN)P molecule shows a higher energy shift (Figure 5c). Since no other absorption is present in the spectra, the solutions are colorless. The electrochemical reduction provokes, generally, a dimming of the main UV peak along with the formation of a low energy broad band, right in the middle of the visible region around 500 nm. In particular, in the case of the derivate iQ-iQ, a strong absorption band centered at 456 nm is present, together with a noticeable decrease in the intensity of the 350 nm UV band. A weak absorption at 594 nm is also present at intermediate potentials, but is subsequently bleached while the reaction proceeds. A similar behavior is observed with the asymmetric compound iQ-Q. Nevertheless, in this case, the absorption band of the reduced form is shifted to higher wavelengths (493 nm), and an incomplete bleaching of the strong band localized at 603 nm takes place. The spectra of the oxidized forms are recovered upon reoxidation, by switching back to the initial potentials. As can be noted from the spectroelectrogram of the iQ-Q compound, a broad weak absorption from 400 to 700 nm can be detected in the visible region. Although the reason for this kind of behavior cannot be clearly identified, a possible explanation might be the formation of stable intermolecular aggregates or dimers. A similar phenomenon has been observed for viologens-based electrochromic devices during ageing tests. Possible causes have been attributed to the radical cation salt deposition on the electrode surface. These deposits might contain spin-paired radical cation dimers with a sandwiched structure, reorientating into ordered phase during open-circuit periods [7,8]. Further investigation on deposited heptyl violognes radicals revealed the presence of a contribution from dimerization of radical cations in solution [9]. A second mechanism for dimer formation can involve a comproportionation reaction between the neutral completely reduced specie (DE^0^) and the oxidized specie (DE^2+^) [10,11]:

DE^0^ + DE^2+^ = (DE_2_^0^)^2+^

The dimer has lower electroactivity as a consequence of its very slow oxidation rate [12], and a decreasing of the write-erase efficiency of the system is thus observed [13]. It has been demonstrated that the use of redox mediators can have a beneficial effect [14] on the functionalization of the nitrogen atoms of the bipyridine core [15,16,17]. In our case, we suppose that a similar aggregation or dimerization process can take place in the solution during spectroelectrochemical measurements, resulting in a loss of electroactivity and a deviation of the observed spectrum from the one expected from the reduced form. The symmetric structure and the higher stability of the intermediate radical cation in Q-Q can have a contribution in making this effect even more evident in this derivative.

In view of its good electrochemical and spectroelectrochemical behavior, iQ-Q was selected for further derivatization with different substituents. The synthetic details are reported in the supporting information, together with the compounds’ coding (Table SI_1). Compound iQ-iQ was not further developed, as the functionalization of its nitrogen atoms proved to be impractical from the synthetic point of view. Derivatives of iQ-Q carrying halogen substituents on the 6-position of the isoquinoline ring (fluorine for derivate FiQ-Q) or on the 7-position of the quinoline ring (Chlorine in iQ-ClQ), and one with a tetramethylene tether connecting the 2- and 3-position of quinoline ring (twQ-iQ), were prepared (Figure 1).

**Figure 6:**
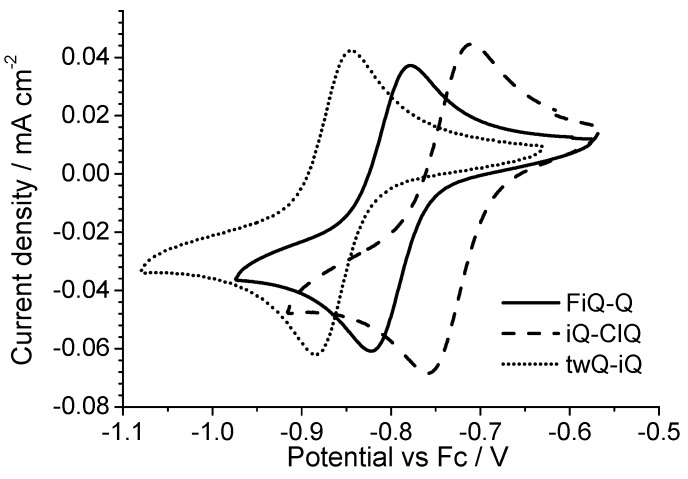
Cyclic voltammograms of derivatives FiQ-Q (F-substituent on the isoquinolinic ring), iQ-ClQ (Cl-substituent on the quinolinic ring), and twQ-iQ.

All of the derivatives show a two-electron reversible electron transfer (Figure 6) with a low peak separation (<40 mV). The Cl- and F-substituted derivatives show a small but significant deviation from the reduction potential measured on the parent compound iQ-Q. The Cl-derivate is slightly easier to reduce, while the F-substituted is not. In the case of the chlorine presence this result is not surprising, even if often the mesomeric effect mitigates the potential shift due to the strong chlorine electron affinity. However, in the case of the small, polarizing fluorine atom, the behavior cannot be rationalized taking in account just the nature of the substituent. Finally, the twQ-iQ (with the tetramethylene moiety connecting 2- and 3-positions on the quinolinic end) is the hardest compound to be reduced, and shows the highest LUMO energy in Table 1. This can be explained as the consequence of the electron-donating effect of the alkyl groups on LUMO energy. From this analysis, it is evident that the chemical features of the substituent groups play an important but not crucial role in driving the electronic (and thus the electrochemical) molecular properties. A more complete scheme can be drawn only considering the effect of the substituent on the molecular structure.

**Figure 7:**
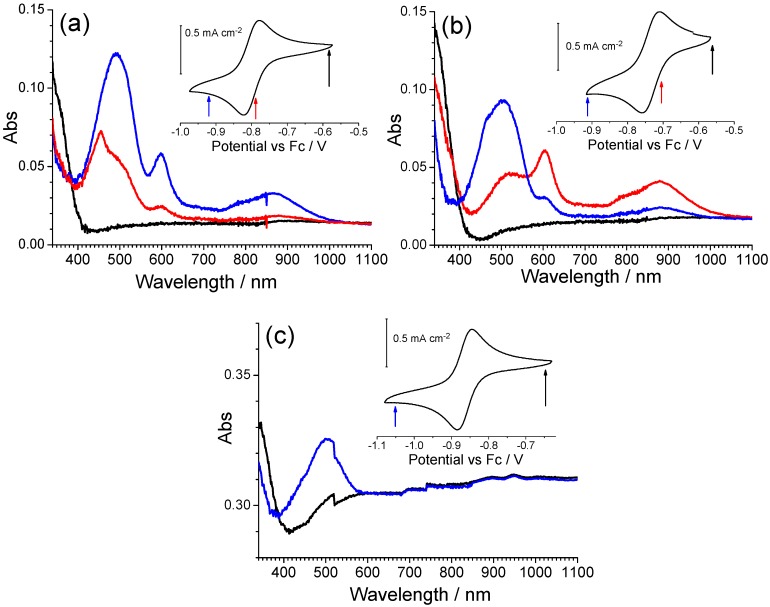
Spectroelectrochemistry of derivatives (**a**) FiQ-Q (F-substituent on the isoquinolinic ring), (**b**) iQ-ClQ (Cl on the quinolinic ring), and (**c**) twQ-iQ (buthylene chain).

Derivatives of the iQ-Q compound were also characterized by spectroelectrochemistry (Figure 7).

In iQ-ClQ (chlorine in the 7-position on the quinolinic end), the absorption bands of the reduced form are centered at 503 and 506 nm, respectively, thus being a little red shifted in comparison with the parent compound. An opposite behavior is observed for compounds FiQ-Q (490 nm) and iQ-twQ (467 nm). In this case, in fact, a blue shift of the band is observed. The effect is more intense in derivative twQ-iQ, demonstrating that the substituent on the 2-3-position of the quinoline ring can strongly alter the electrochemical and optical properties of the compound. A closer look at the spectroelectrochemistry of compounds FiQ-Q and iQ-ClQ reveals that the reduction process is associated with the formation of a third species, absorbing around 455–460 nm. The extra absorption is not bleached upon reoxidation, a behavior similar to the one previously observed for compound Q-Q. As we already discussed before, this phenomenon can be ascribed to the formation of radical-cation dimers with low electroactivity.

### 2.3. Ab Initio Calculations

**Figure 8:**
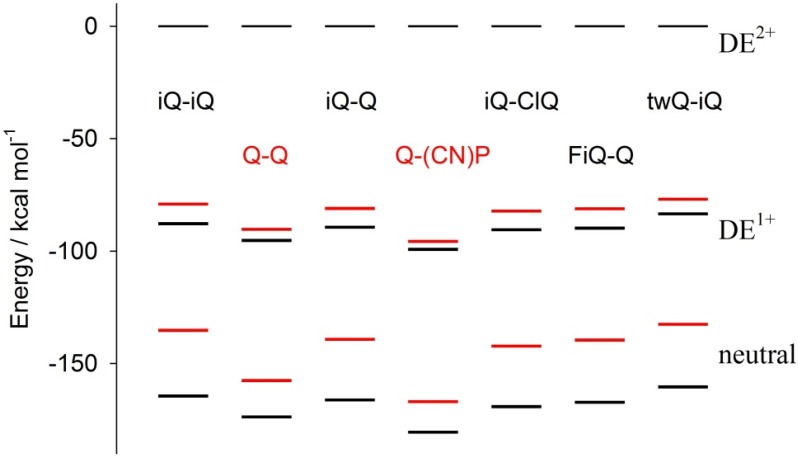
Calculated energy of the cations and neutral molecules with respect to the energy of the dication parent species (assumed to be zero). The black and red bars correspond to the optimized or dication quenched geometry, respectively. The data were obtained at the B3LYP/6-31G(d) level of the theory.

Figure 2 shows that the DE^2+^ and DE^0^ species are characterized by a completely different electronic structure. Indeed, the parent dicationic species features two rigid and planar azinium endcapping units connected to the central ethenylic bridge by C-C carbon bonds. This arrangement is connected with a certain rotational freedom, thus leading to a severely distorted ground state. In contrast, the neutral reduced species features a quinoid electronic structure showing only one rotational degree of freedom in the bridge connecting the two heterocycles. In principle, such a structure allows for smaller deviations from planarity. Thus, the variation of geometrical structural factors at the oxidation state is expected to play a major role. Figure 8 shows the molecular total electronic energy of all of the compounds as a function of the oxidation state and calculated following a full optimization procedure, which implies a relaxation of the molecular geometry (black bar) or the estimated maintaining of the geometry fixed (red bar) to the geometry of the original parent DE^2+^ species (the energy of the DE^2+^ parent species is set as the conventional zero energy). This investigation gives a direct insight into the incidence of redox-induced geometrical distortion as a function of the connectivity and substitution pattern of the specific investigated compound. Moreover, as the investigation was carried out as a function of the oxidation state, the study gives insight on the role of geometrical factors on the relative stabilization of monocation over dication.

Note that the energy differences between the vertical and relaxed structures of the DE^1+^ states of the Q-Q and Q-(CN)P compounds, i.e. the distance between the red and black bars, is about 50% of those found for all of the other compounds; the same holds for the neutral species. Thus, the diazinium ethenes here investigated can be grouped into two families: (i) the Q-Q and Q-(CN)P compounds featuring a virtually planar geometry; and (ii) the remaining compounds, where the intramolecular reciprocal ring-to-ring disposition is significantly “distorted”, showing a ring-to-ring dihedral angle in the 60° to 90° range (Table 2). It is noteworthy that the value of the aforementioned dihedral angle decreases with the oxidation state. In this respect, Q-Q and Q-(CN)P again show a peculiar behavior, indeed, the Q-Q compound’s calculated dihedral angles are 0°, 25.3°, and 0° for oxidation states of 2^+^, 1^+^, and 0, respectively, while Q-(CN)P features a flat geometrical disposition invariant with the oxidation state. Remarkably, the structure of the final product neutral species is the most planar of all of the compounds. On the whole, a substantial difference in the electrochemical results can be expected due to the interplay between the benzenoid/quinoid resonance stabilization, as well as to steric effects inducing large differences in the geometry relaxation energies of the parent compound.

From the theoretical point of view, the experimental electrochemical behaviour of the diazinium ethenes (DE) derivatives here investigated is assumed to be represented as two successive single-step reversible reduction processes [18,19].

The relevant elementary step is the first reduction process:

DE^2+^ + e^−^ = DE^1+^

characterized by a redox potential E_2_ (experimental values in Table 1) and the corresponding electron affinity EA(I), and the second reduction process:

DE^1+^ + e^−^ = DE^0^

characterized by a redox potential E_1_ (experimental value in Table 1) and the corresponding electron affinity EA (II).

**Figure 9:**
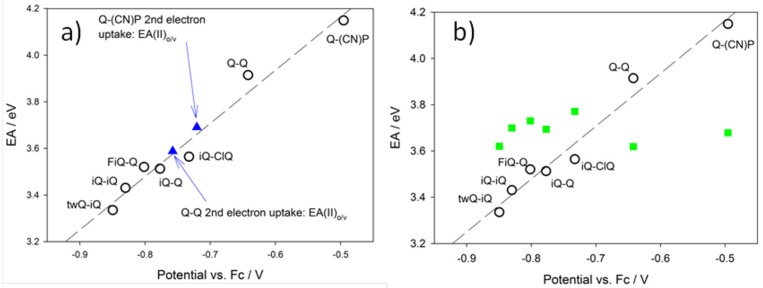
(**a**) Calculated electron affinity (EA) vs. Experimental Reduction Potential. Black circles: vertical electron affinity vs. experimental reduction potential (EA(I)_vert_ vs. E_2_). Blue solid triangles: Q-Q and Q-(CN)P compounds only, which show the relevant EA(II)_o⁄v_ vs. E_1_ (Table 1). (**b**) Calculated EA vs. Experimental Reduction Potential. Black circles: vertical electron affinity vs. experimental reduction potential (EA(I)_vert_ vs. E_2_). Green squares: EA(II)_o⁄v_ vs. E_2_ data.

Figure 9 shows theoretical Electron Affinity (“EA”) values as a function of the experimental potential. Theoretical EAs were calculated using a number of different strategies, aiming at the rationalization of the most peculiar feature of the redox processes investigated: a single two-electron redox wave versus two distinct waves, each of one with a single-electron character [18,20]. Figure 9a reports the EA data calculated, allowing, or not allowing, for structural relaxation (geometry rearrangement as a function of the various oxidation states). The black circles in Figure 9a are representative of the so-called “vertical electron affinity” vs. first process experimental reduction potential (compare E_2_ in Table 1). In this case, EA(I)_vert_ is calculated as:

EA(I)_vert_ = −[Energy(DE^1+^)_vert_ − Energy (DE^2+^)_opt_]

where the subscript “opt” refers to the DE^2+^ species calculated at the fully optimized geometry, whilst the subscript “vert” indicates that the energy, in this case of the DE^1+^ species, is calculated at the same geometry of the parent DE^2+^ species (i.e., with the assumption that the charge transfer process is much faster than the nuclear rearrangement time scale). Thus, the first electron uptake is considered as an instantaneous process, so fast that the relaxation process involving the DE^1+^ species does not affect the reduction potential (this is the opposite of an adiabatic reduction process, where the geometry relaxation of the species involved in the redox process must be taken into account [18]). Note that a correct physical slope is obtained in Figure 9; that is, larger EA values imply less negative reduction potentials. The application of the same principle to the second reduction process (obviously, for the case of Q-Q and Q-(CN)P alone) leads to:

EA(II)_vert_ = −[Energy(DE^0^)_vert_ − Energy (DE^1+^)_vert_]

the values obtained for Q-(CN)P and Q-Q are 3.09 and 2.92 eV, respectively, which clearly do not fit the EA(I)_vert_ vs. E_2_ correlation, shown as the black dashed line in Figure 9 (in fact, the E_1_ values for Q-(CN)P and Q-Q, compare Table 1, are −0.70 and −0.74 V, respectively). Conversely, if we take into account the structural relaxation process coupled with the second charge transfer process, shown as the blue solid triangles in Figure 9a, we obtain again a quite satisfactory correlation with all of the other data. In this case, the relevant equation is:

EA(II)_o/v_ = −[Energy(DE^0^)_opt_ − Energy (DE^1+^)_vert_]

where: Energy (DE°)^0^ is the energy relevant to the neutral relaxed (optimized geometry) species.

As a result, by a comparison of the experimental and theoretical results, we can infer that the first electron uptake can be considered as a vertical/non-adiabatic process where the species involved in the reduction process does not relax to the relevant cation (DE^1+^)-relaxed state. Remarkably, a unique linear pattern is fitted by EA(I)_vert_ vs. E_2_ for all of the compounds, regardless of the presence or not of a second distinct redox wave. The EA(II) values for Q-Q and Q-(CN)P fall on the same line, only if the relaxation of the reduced species to the equilibrium geometries are taken into account (thus, only if EA(II)_o/v_ instead of EA(II)_vert_ values are used). Figure 9b shows the EA(II)_o/v_ values (green squares) plotted against the E_2_ data for all compounds displaying only one two-electron redox wave. The EA(II)_o⁄v_ values are consistently larger than the EA(I)_vert_ ones, and are not linearly correlated with the E_2_ data. This would imply that at the potential of the first reduction, also the uptake of the second electron occurs. Conversely, the EA(II)_o⁄v_ values of Q-Q and Q-(CN)P are too low to allow for the second simultaneous electron uptake to occur (compare the green squares in Figure 9b). This is likely the rationale for the observation of two single electron steps for such compounds. Dynamical effects seem to play a major role, as already found in reduction processes involving complex systems [21,22]. Thus, the initial substantial differences (found in the parent DE^2+^ species) in the reciprocal orientation of quinolinic rings of flat (Q-Q and Q-(CN)P) to orthogonal orientation (all remaining compounds save Q-Q and Q-(CN)P), play a major role in determining a qualitative difference in the electrochemical reduction process. Note also that the regular variation in the carbon−carbon bond distance going from the DE^2+^ to the DE^0^ species, (Table SI_1). The C=C distance in the central moiety changes from 1.35 Å to 1.438 Å, and, on average, the opposite is found for the C-Benzene ring distance. This variation in the C-C distances accounts for a change from aromatic to quinoid character in the cyclic π-systems, going from the oxidized to the reduced neutral species (Figure 2).

## 3. Discussion

The electronic (and thus the electrochemical) properties strongly depend on the molecular structure. Energy levels, indeed, are influenced by the electron donor/acceptor nature of the molecular fragments, as well as by the molecular geometry, which in turn depends on the substituents. The geometry can be rationalized in terms of the planarity of the different dication molecules, which is expressed as the dihedral angle between the two aromatic rings (quinoline–quinoline, quinoline–isoquinoline, isoquinoline–isoquiline, and quinoline–pyridine) of the substitute ethenes. Generally, neutral molecules are more planar than dicationic species, and, indeed, their dihedral angles are lower (see Table 2). The exceptions are the symmetric Q-Q and the asymmetric Q-(CN)P ethenes, which are also the only molecules to show two mono-electronic reduction waves instead of a single two-electron reduction one. Therefore, the two effects (nature of substituent and its influence on the molecular structure) can be applied to rationalize the LUMO energy levels of the dications, and thus their reduction potentials. First of all, as already pointed out, the isoquinoline moiety has higher electron-withdrawing effect with respect to the quinoline one; however, the large difference of the reduction potentials (200 mV) between the parent compounds (Q-Q and iQ-iQ) can be also explained taking into account the large dihedral angle of the iQ-iQ^2+^ cation compared to the planar Q-Q^2+^. The asymmetric iQ-Q^2+^ compounds have a LUMO level which lies at an intermediate value with respect to the parent symmetric systems; however, due also to its large dihedral angle, the value is closer to iQ-iQ (the difference is 70 mV) rather than to Q-Q (where the difference is 130 mV). The geometrical features of the fluorine-substituted dication (FiQ-Q) are very similar to those of the iQ-Q^2+^, and thus are the corresponding energy levels. Despite the strong electron withdrawing effect of the substituent, the slight distortion in the neutral FiQ-Q molecule may also play a role. The iQ-ClQ^2+^ molecule has a lower dihedral angle, and considering the dual nature of the substituent (Cl^-^ is electron donating according to mesomeric p-donation and electron withdrawing according to inductive effects) its reduction potential is higher than those of the iQ-Q and FiQ-Q dications. TwQ-iQ and Q-(CN)P show the lowest and the highest reduction potential, respectively, i.e., the distorted, electron-rich unsaturated ring substituent in twQ-iQ pushes the LUMO level to the highest energy value, while the planar, strong electron-withdrawing cyano-pyridinic group lowers the LUMO level, leading it to be the most prone to reduction dication specie. Both compounds can be regarded as opposite products in terms of molecular design in the series of our ethenes.

## 4. Materials and Methods

All of the derivatives have been prepared though the adaptation of procedures known in the literature. Details as well as the identity and purity characterizations for the new compounds are given in the Supporting Information section.

Electrochemical measures were carried out using a galvanostat-potentiostat in a single chambered, three-electrode electrochemical cell. All of the electrochemical tests were performed inside a glove box filled with argon ([O_2_] < 1 ppm) at room temperature ranging from 21 to 23 °C. Glassy Carbon (GC) and gold discs were used as working electrodes. The counter and pseudo-reference electrodes were a Pt foil and a Ag/AgCl wire, respectively. Both the GC and gold pins were well polished with an alumina 0.1 μm suspension, sonicated for 15 min in acetone, and rinsed with 2-propanol before use. The Ag/AgCl pseudo-reference electrode was calibrated before and after each measurement using a 1 mM ferrocene solution in the electrolyte (0.1 M solution of TBAPF_6_ in acetonitrile); no more than 5mV difference was observed between two successive calibrations. The coefficient of variation (CV) scans were repeated three times (three cycles); the second and the third cycle did not show significant differences. The second cycle is reported in the present manuscript. Optical absorption spectroscopy was performed using a UV-visible spectrophotometer with extended range up to 1100 nm. The spectroelectrochemical measurements of the solutions were performed using a thin layer quartz cell with 0.5 or 1 mm optical path lengths, and using gold mesh, Pt wire, and Ag/AgCl as the working, counter, and reference electrodes, respectively.

Ab initio molecular orbital calculations were performed using the Gaussian 03 suite of programs. Screening full optimization geometry calculations, aiming at comparing the B3LYP/3-21G* and B3LYP6-31G(d) results, were performed in a vacuum without any symmetry constraints. The geometries optimized in the gas phase were used to perform the solvation energy calculation of the various species involved in the determination of the Electron Affinity. The solvation energies were obtained by using the Barone and Cossi’s polarizable conductor model (CPCM) method, which is based on the Polarized Continuum Model (PCM) of Tomasi. Acetonitrile is the solvent to be simulated; to this purpose, the default solvent parameters are used as implemented in Gaussian 03. In particular, the values used were: solvent radius RSolv = 2.1550 Å, dielectric constant Eps = 36.64, and Epsinf = 1.8060, which are the static and dynamic dielectric constant values. The energies of solvated species were estimated at the B3LYP/-31G(d)//B3LYP/6-31G(d) level of the theory. The vertical electronic excitation energies were calculated using time-dependent density functional theory (TD-DFT) at the B3LYP/6-31G(d) level of the theory.

## 5. Conclusions

A large class of 1,2-diazinium-ethenes based on quinoline and isoquinoline moieties was prepared and characterized. These compounds show peculiar absorption properties, which changes upon reduction and can be used in electro-optic applications. In particular, the two iQ-Q system shows a reversible bielectronic reduction from the dication to the neutral state, which combines with the bleaching of the visible absorption band. Electrochemistry was used to determine the energy levels, and in particular, the LUMO was tuned over a range of 0.4 eV by the design of proper molecular structures. The substituent effect on the molecular features was also rationalized by computational analysis. Such a tool gave insight on the structure–property relationships, particularly regarding to the mono- versus two-electron nature of the redox steps involved. Finally, in situ spectroelectrochemistry measurements demonstrated that all compounds undergo a complete bleaching of the visible absorption of the neutral forms upon oxidation, a very relevant and demanding characteristic that is particularly appreciated for all see-through applications of electrochromic materials and devices (smart windows, sunglasses, domestic appliances).

## Figures and Tables

**Table 1:** Electrochemical data obtained from cyclic voltammograms.

Derivate	E_2_/V	E_1_/V	LUMO/eV
Q-Q	−0.62 ^1^	−0.74^1^	−4.6
iQ-iQ	−0.83		−4.4
Q-(CN)P	−0.49	−0.72	−4.7
iQ-Q	−0.76		−4.4
FiQ-Q	−0.80		−4.4
iQ-ClQ	−0.73		−4.5
twQ-iQ	−0.86		−4.3

^1^ Data obtained from DPV (see Figure SI_1).

**Table 2:** Dihedral angles between rings obtained from calculations. The data were obtained at the B3LYP/6-31G(d) level of the theory.

Derivate	Dication/°	Monocation/°	Neutral/°
Q-Q	0.0	25.3	8.2
iQ-iQ	79.4	30.2	18.2
Q-(CN)P	0.0	0.0	0.0
iQ-Q	89.3	30.8	0
FiQ-Q	89.1	31.0	17.6
iQ-ClQ	57.0	31.2	26.8
twQ-iQ	72.5	58.3	29.5

## Supplementary Materials

Synthetic details and Table SI_1 are available online at www.mdpi.com/1996-1944/10/7/802/s1.

## References

[1] Beverina L., Pagani G.A., Sassi M. (2014). Multichromophoric electrochromic polymers: colour tuning of conjugated polymers through the side chain functionalization approach. Chem. Comm..

[2] Sassi M., Salamone M.M., Ruffo R., Patriarca G.E., Mari C.M., Pagani G.A., Posset U., Beverina L. (2016). State-of-the-art neutral tint multichromophoric polymers for high-Contrast see-through electrochromic devices. Adv. Funct. Mater..

[3] Mortimer R.J. (1999). Organic electrochromic materials. Electrochim. Acta.

[4] Hünig S., Kemmer M., Wenner H., Barbosa F., Gscheidt G., Perepichka I., Bäuerle P., Emge A., Peters K. (2000). Violene/Cyanine hybrids as electrochromics part 2: tetrakis(4-dimethylaminophenyl)ethene and its derivatives. Chem. Eur. J..

[5] Hünig S., Berneth H. (1980). Two step reversible redox systems of the weitz type. Organic Chemistry. Topics in Current Chemistry.

[6] Michaelis L. (1935). Semiquinones, the intermediate steps of reversible organic oxidation-reduction. Chem. Rev..

[7] Compton R.G., Waller A.M., Monk P.M.S., Rosseinsky D.R. (1990). Electron paramagnetic resonance spectroscopy of electrodeposited species from solutions of 1,1′-bis-(p-cyanophenyl)-4,4′-bipyridilium (cyanophenyl paraquat, CPQ). J. Chem. Soc. Faraday Trans..

[8] Barna G.G. (1980). The Morphology of viologen films on transparent oxide electrodes. J. Electrochem. Soc..

[9] Goldon A., Przyluski J. (1985). Studies of electrochemical properties of N-heptylviologen bromide layers. Electrochim. Acta.

[10] Monk P.M.S., Fairweather R.D., Ingram M.D., Duffy J.A. (1992). Evidence for the product of the viologen comproportionation reaction being a spin-paired radical cation dimer. J. Chem. Soc. Perkin Trans..

[11] Monk P.M.S., Hodgkinson N.M., Ramzan S.A. (1999). Spin pairing (‘dimerisation’) of the viologen radical cation: kinetics and equilibria. Dyes Pigment.

[12] Rosseinsky D.R., Monk P.M.S. (1990). Kinetics of comproportionation of the bipyridilium salt p-cyanophenyl paraquat in propylene carbonate studied by rotating ring–disc electrodes. J. Chem. Soc. Faraday Trans..

[13] Poizat O., Sourisseau C., Corset J. (1986). Vibrational and electronic study of the methyl viologen radical cation MV+. in the solid state. J. Mol. Struct..

[14] Yasuda A., Mori H., Takehana Y., Ohkoshi A., Kamiya N. (1984). Electrochromic properties of the n-heptyl viologen-ferrocyanide system. J. Appl. Electrochem..

[15] Barna G.G., Fish J.G. (1981). An improved electrochromic display using an asymmetrical viologen. J. Electrochem. Soc..

[16] Bruinink J., Kregting C.G.A., Ponjeé J.J. (1977). Modified viologens with improved electrochemical properties for display applications. J. Electrochem. Soc..

[17] Barltrop J.A., Jackson A.C. (1984). The synthesis and electrochemical study of new electrochromic viologen-based materials. J. Electrochem. Soc. Perkin Trans..

[18] Bruno C., Paolucci F., Marcaccio M., Benassi R., Fontanesi C., Mucci A., Parenti F., Preti L., Schenetti L., Vanossi D. (2010). Experimental and theoretical study of the p- and n-doped states of alkylsulfanyl octithiophenes. J. Phys. Chem. B.

[19] Tassinari F., Vanossi D., Mucci A., Parenti F., Fontanesi C. (2013). Regiochemistry in the electrochemical assisted grafting of glassy carbon. with focus on sterical hindrance of lateral chains in the electroreduction process of multi-functionalized bithiophene. J. Electroanal. Chem..

[20] Winget P., Weber E.J., Cramer C.J., Truhlar D.G. (2000). Computational electrochemistry: aqueous one-electron oxidation potentials for substituted anilines. Phys. Chem. Chem. Phys..

[21] Cigarini L., Vanossi D., Bondioli F., Fontanesi C. (2015). A novel synthetic strategy for magnetite-type compounds. A combined experimental and DFT-computational study. Phys Chem Chem Phys..

[22] Fontanesi C., Bortolotti C.A., Vanossi D., Marcaccio M. (2011). Dissociation dynamics of asymmetric alkynyl(aryl)iodonium radicals: an ab initio DRC approach to predict the surface functionalization selectivity. J. Phys. Chem. A.

