# Supplementary material for: An Integrated Theoretical/Experimental Study of Quinolinic–Isoquinolinic Derivatives Acting as Reversible Electrochromes
Source: Materials (Basel). 2017 Jul 15;10(7):802. doi: 10.3390/ma10070802 (PMC5551845; doi:10.3390/ma10070802)
Supplement: Supplementary file 1 [file materials-10-00802-s001.pdf]

An integrated theoretical/experimental study of quinolinic-isoquinolinic derivatives acting as reversible electrochromes

Mauro Sassi[a], Matteo M. Salamone[a], Luca Beverina[a], Gianluca Longoni[a], Claudio M. Mari[a], Claudio Fontanesi[b], Davide Vanossi[b], Luigi Cigarini[b], Riccardo Ruffo[a]

[a]Dipartimento di Scienza dei Materiali, Università degli Studi di Milano Bicocca, via Cozzi 55, 20125 Milano, Italy

[b]Dipartimento di Ingegneria Enzo Ferrari, Università degli Studi di Modena e Reggio Emilia, via Vivarelli 10, 41125 Modena Italy

Supplementary Materials

1. Synthesis of Molecules

DERIVATE **7, Q-Q**

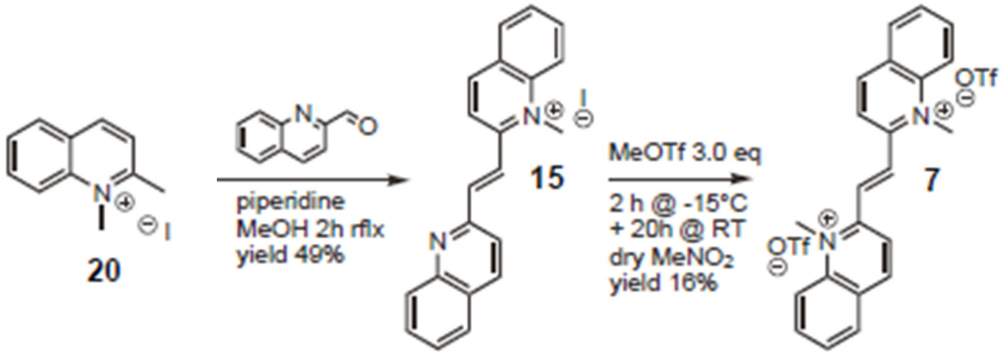

Synthesis of 1,2-dimethylquinolinium iodide (**20**)

A mixture of 2-methylquinoline (1.000 g, 6.98 mmol), MeI (3.960 g, 27.9 mmol) was heated in a CEM Discover microwave reactor for 8 min (P max =20 W, 140 °C), and cooled to room temperature (RT). The pale yellow solid product was washed with toluene and filtered, washing with more toluene and then with Et_2O. Residual solvent was evaporated under reduced pressure (0.4 torr) at 40 °C (1.452 g, 5.09 mmol, yield 73%, m.p.: 197 ◦ C (dec.)).

^{1}H NMR (500 MHz, DMSO−d_6) δ [ppm]: 9.10 (d, J=8.5 Hz, 1H), 8.60 (d, J=9.0 Hz, 1H), 8.40 (d, J=8.1 Hz, 1H), 8.24 (t, J=8.13 Hz, 1H), 8.12 (d, J=8.5 Hz, 1H), 8.00 (t, J=7.7 , 1H), 4.45 (s, 3H), 3.08 (s, 3H).

Synthesis of (E)-1-methyl-2-(2-(quinolin-2-yl)vinyl)quinolinium iodide (**15**)

A mixture of 20 (824 mg, 2.89 mmol), quinoline-2-carboxyaldehyde (500 mg, 3.18 mmol) and piperidine (2 drops) in MeOH (8 ml) was heated to reflux for 2 h. The solution was cooled with an ice-water bath obtaining an orange precipitate, which was filtered and washed with cold MeOH and Et_2O. Residual solvent was removed under reduced pressure at 50 ◦ C (596 mg, 1.405 mmol, yield 49%, m.p.: 220 ◦ C (dec.)).

^{1}H NMR (500 MHz, DMSO−d_6) δ [ppm]: 9.22 (d, J=8.8 Hz, 1H), 8.71 (d, J=8.8 Hz, 1H), 8.66 (d, J=9.0 Hz, 1H), 8.59 (d, J=8.5 Hz, 1H), 8.46-8.43 (m, 2H), 8.30-8.23 (m, 3H), 8.14 (d, J=8.5 Hz, 1H), 8.08 (d, J=8.1 Hz, 1H), 8.04 (t, J=7.6 Hz, 1H), 7.87 (td, J=7.0 Hz, J=1.2 Hz, 1H), 7.71 (t, J=7.5 Hz, 1H), 4.67 (s, 3H); 13 C NMR (125.7 MHz, DMSO−d_6) δ [ppm]: 156.05, 153.60, 148.11, 145.64, 145.61, 139.75, 137.85, 135.86, 131.06, 130.70, 129.96, 129.72, 128.88, 128.54, 128.46, 128.40, 125.16, 122.38, 121.99, 119.96, 40.96.

Synthesis of (E)-1-methyl-4-(2-(1-methylquinolinium-2-yl)vinyl)-quinolinium trifluoromethanesulfonate (**7**)

Under N_2 atmosphere, MeOTf (464 mg, 2.83 mmol) was added to a stirred suspension of 15 (400 mg, 0.94 mmol) in dry $MeNO_2$ (4 ml) cooled to −15 ◦ C with an ice bath. The mixture was kept under stirring at −15 ◦ C for 2 h, and stirred overnight at room temperature. After a 15 min, a clear solution was obtained. More product was precipitated after the addition of Et_2O, collected by filtration, dried in a vacuum, and crystallized twice from EtOH (91 mg, 0.149 mmol, yield 16%, m.p.: 287-288 ◦ C).

^{1}H NMR (500 MHz, CD_3CN) δ [ppm]: 9.20 (d, J=8.6 Hz, 2H), 8.53 (d, J=9.0 Hz, 2H), 8.44 (dd, J=8.2 Hz, J=1.1 Hz, 2H), 8.42 (J=8.6 Hz, 2H), 8.34 (t, J=8.12 Hz, 2H), 8.18 (s, 2H), 8.10 (t, J=7.4 Hz, 2H), 4.62 (s, 6H); ^{13}C NMR (125.7 MHz, CD_3CN) δ [ppm]: 153.97, 147.20, 140.00, 136.74, 134.94, 130.83, 130.65, 129.78, 122.98, 119.19, 41.34.

DERIVATIVE **4 iQ-Q**

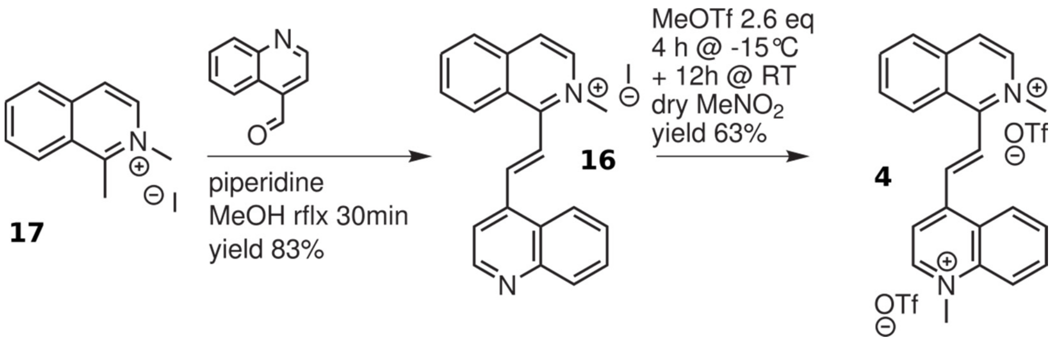

Synthesis of 1,2-dimethylisoquinolinium iodide (**17**)

A mixture of 1-methylisoquinoline (5.00 g, 34.9 mmol), MeI (5.95 g, 41.9 mmol) and 7 ml of acetone was heated in a CEM Discover microwave reactor for 8 min (P max =50 W, 110 °C), and cooled to RT. The white solid product was washed with acetone and filtered, washed with more acetone and then with Et_2O. Residual solvent was evaporated under reduced pressure (0.4 torr) at 40 ◦ C (8.72 g, 30.6 mmol, yield 88%, m.p.:210-211 ◦ C).

1 H NMR (500 MHz, DMSO−d_6) δ [ppm]: 8.79 (d, J=8.7 Hz, 1H), 8.70 (d, J=6.9 Hz, 1H), 8.40 (d, J=6.9 Hz, 1H), 8.31 (d, J=8.1 Hz, 1H), 8.22 (t, J=7.7 Hz, 1H), 8.05 (t, J=7.7 Hz, 1H), 4.40 (s, 3H), 3.22 (s, 3H).

Synthesis of (E)-2-methyl-1-(2-(quinolin-4-yl)vinyl)isoquinolinium iodide (**16**)

To a mixture of 17 (5.000 g, 17.53 mmol) and quinoline-4-carboxyaldehyde (3.113 g, 19.80 mmol), a solution of piperidine (0.50 ml, 430 mg, 5.0 mmol) in 10 ml of MeOH was added. The mixture was heated to reflux for 30 min, obtaining a solid. The mixture was allowed to cool to room temperature and 25 ml of MeOH was added. The solid was filtered and washed with MeOH and Et_2O. Residual solvent was removed under reduced pressure at 50 °C. Product was recovered as a pale yellow solid (6.165 g, 14.53 mmol, yield 83%, m.p.: 217-218 °C (dec.)).

^{1}H NMR (500 MHz, DMSO−d_6) δ [ppm]: 9.12 (d, J=4.5 Hz, 1H), 8.87 (d, J=6.9 Hz, 1H), 8.69 (d, J=8.7 Hz, 1H), 8.60 (d, J=6.8 Hz, 1H), 8.41 (d, J=8.2 Hz, 1H), 8.33 (d, J=8.2 Hz, 1H), 8.31-8.27 (m, 2H), 8.18 (d, J=4.5 Hz, 1H), 8.15 (d, J=8.1 Hz, 1H), 8.10-8.04 (m, 2H), 7.87 (t, J=7.7 Hz, 1H), 7.71 (t, J=7.6 Hz, 1H), 4.50 (s, 3H). ^{13}C NMR (125.7 MHz, DMSO−d 6) δ [ppm]: 157.15, 150.97, 148.73, 140.13, 140.02, 137.85, 137.22, 136.62, 131.88, 130.38, 130.18, 130.14, 128.20, 127.88, 127.52, 126.01, 125.13, 124.42, 123.80, 119.03, 47.41.

Synthesis of (E)-1-methyl-4-(2-(2-methylisoquinolinium-1-yl)vinyl)-quinolinium trifluoromethanesulfonate (**4**)

Under N_2 atmosphere, MeOTf (200 mg, 1.219 mmol) was added to a stirred suspension of 16 (200 mg, 0.471 mmol) in dry MeNO 2 (7 ml) cooled to −15 ◦ C with an ice/salt bath. The mixture was kept under stirring at −15 ◦ C for 4 h obtaining a clear pale yellow solution, and stirred overnight at room temperature. Product was precipitated as a pale yellow solid after addition of Et_2O, collected by filtration, and dried in a vacuum (280 mg, 0.458 mmol, yield 97%). ^{1}H NMR (500 MHz, DMSO−d_6) δ [ppm]: 9.68 (d, J=6.3 Hz, 1H), 8.92 (d, J=6.9 Hz, 1H), 8.78 (d, J=6.2 Hz, 1H), 8.71-8.66 (m, 3H), 8.62 (d, J=8.9 Hz, 1H), 8.47-8.43 (m, 2H), 8.38-8.30 (m, 3H), 8.13-8.09 (m, 2H), 4.72 (s, 3H), 4.30 (s, 3H); ^{13}C NMR (125.7 MHz, DMSO−d 6) δ [ppm]: 156.02, 150.22, 149.97, 139.17, 137.91, 137.42, 137.27, 136.86, 135.93, 132.00, 130.74, 130.03, 129.63, 128.27, 127.37, 127.33, 126.83, 125.77, 120.28, 119.83, 47.46, 46.04; Anal. Calcd for $C_{24}H_{20}F_6N_2O_6S_2$: C, 47.21; H, 3.30; N, 4.59; S, 10.50. Found: C, 47.30; H, 3.72; N, 4.61; S, 10.44.

DERIVATIVE **5, iQ-iQ**

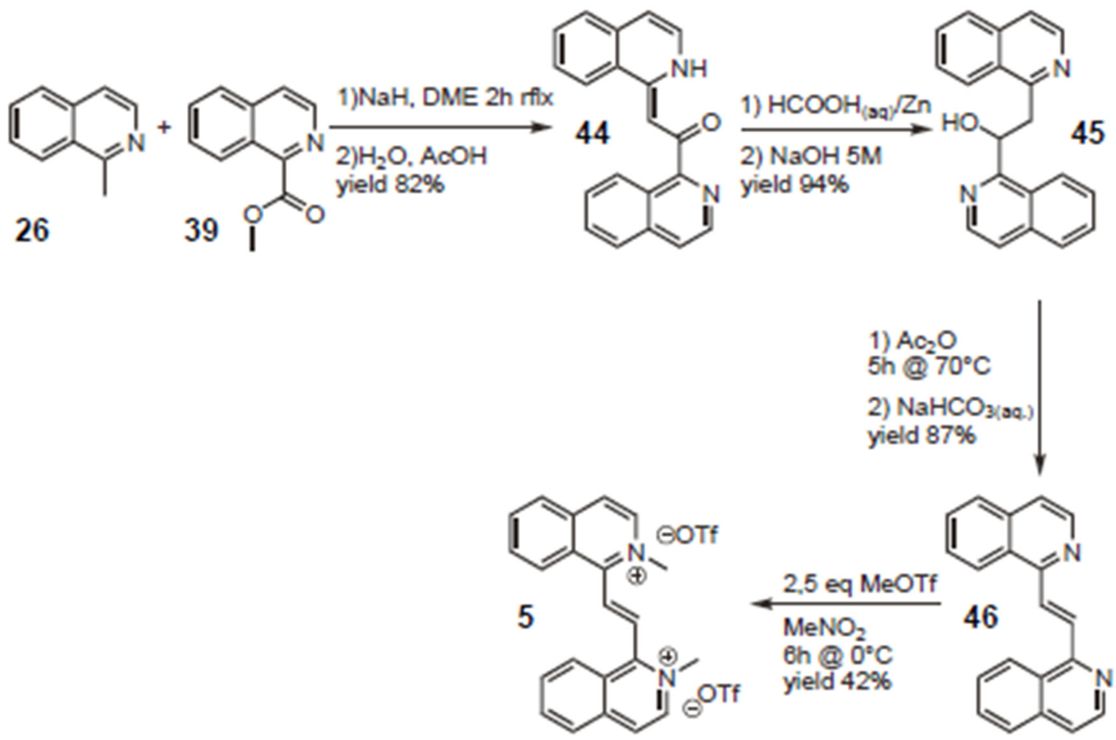

Synthesis of methyl isoquinoline-1-carboxylate (**39**)

In a 250 ml RBF a suspension of isoquinoline-1-carboxylic acid (10.726 g, 61.9 mmol) in 75 ml of MeOH was prepared. The mixture was cooled with an ice-water bath, and concentrated H_2SO_4 (24.88 g, 253 mmol) was slowly added under stirring. The mixture was stirred at room temperature for 10 min and transferred in a CEM 35 ml microwave pressure vessel (4 aliquots were used) and heated at 105 °C, 30 W for 1 h. The mixture was cooled to RT, slowly poured in water (300 ml) and basified to pH 10 by addition of Na_2CO_3. The mixture was extracted with 60 ml+2 × 30ml of CH_2Cl_2 collecting the organic phase and drying over Na_2SO_4. Product was recovered after solvent evaporation under reduced pressure and purified by vacuum distillation (b.p.: 130 ◦ C at 5 mmHg). Clear oil (8.110 g, 43.3 mmol, yield 70%).

^1H NMR (500 MHz, $CDCl_3$) δ [ppm]: 8.83 (d, J=8.5 Hz, 1H), 8.63 (d, J=5.5 Hz, 1H), 7.88 (d, J=8.1 Hz, 1H), 7.83 (d, J=5.5 Hz, 1H), 7.74 (t, J=8.1 Hz, 1H), 7.69 (t, J=7.8 Hz, 1H), 4.10 (s, 3H).

Synthesis of (Z)-2-(isoquinolin-1(2H)-ylidene)-1-(isoquinolin-1-yl)ethanone (**44**)

Under N_2 atmosphere, in an oven dried 250 ml 3-necked RBF, fitted with reflux condenser, septum and dropping funnel, a suspension of NaH (1.406 g, 58.6 mmol) in anhydrous DME was prepared. A solution of 1-methylisoquinoline (2.00 g, 14.0 mmol) and **39** (3.14 g, 16.8 mmol) in 20 ml of anhydrous DME was dropwise added to the refluxing suspension of NaH. The suspension turned to yellow and after 1 h a brown homogeneous solution was obtained. After additional 2 h the mixture was cooled with an ice-water bath, and Et_2O (25 ml) was added followed by AcOH(1.74 g). After 5 min H_2O (18 ml) was added followed by more AcOH (1.74 g). The mixture was kept under stirring at RT for 1 h. The bright yellow solid was filtered on a Büchner funnel washing with Et_2O. The filtrate was extracted with 3 × 20ml of Et_2O collecting the organic phase and washing it with 10 ml of $NaHCO_3$ (aq). The organic phase was dried over Na_2SO_4 and the volume was reduced to 5 ml under reduced pressure. The precipitate was filtered and collected. Product from the first filtration was continuously extracted with CH_2Cl_2 in a Soxhlet apparatus to remove insoluble impurities and recovered after solvent evaporation under reduced pressure. Bright yellow powder (3.399 g overall, 11.39 mmol, yield 82%, m.p.: 175.5-177.0 ◦ C).

^1H NMR (500 MHz, C 6 D 6) δ [ppm]: 16.42 (s, 1H), 9.88 (d, J=8.6 Hz, 1H), 8.75 (d, J=5.5 Hz, 1H), 8.02 (d, J=8.3 Hz, 1H), 7.78 (s, 1H), 7.56 (d, J=8.2 Hz, 1H), 7.51 (td, J=7.2 Hz, J=1.3 Hz, 1H), 7.41-7.38 (m, 2H), 7.20 (t, J=7.9 Hz, 1H), 7.08 (d, J=7.9 Hz, 1H), 7.04 (t, J=7.6

Hz, 1H), 6.48 (t, J=5.8 HZ, 1H), 6.17 (d, J=6.7 Hz, 1H); ^{13}C NMR (125.7 MHz, DMSO−d 6) δ [ppm]: 186.23, 158.17, 153.84, 141.91, 137.10, 135.87, 133.10, 130.84, 129.50, 128.50, 128.26, 127.78, 127.70, 127.60, 125.94, 125.38, 124.27, 122.47, 111.66, 88.11.

Synthesis of 2-(isoquinolin-1-yl)-1-(quinolin-4-yl)ethanol (**45**)

To a stirred solution of **44** (2.000 g, 6.70 mmol) in aqueous formic acid 50% (100 ml), zinc dust (2.091 g, 32.0 mmol) was added. The solution rapidly changed its colour from red to pale yellow. The mixture was kept under stirring for 25 min and filtered collecting the filtrate in an ice-cooled stirred 5 M NaOH solution (250 ml). A white solid separated and the mixture was kept under stirring for 15 min, filtered and washed with 30 ml of 5 M NaOH solution followed by 30 ml of deionized water. The white solid was dried in an evacuated desiccator (1.890 g, 6.29 mmol, yield 94%, m.p.: 167.7-168.5 ◦ C).

^{1}H NMR (500 MHz, DMSO−d_6) δ[ppm]: 8.57 (d, J=8.44 Hz, 1H), 8.40 (d, J=5.6 Hz, 1H), 8.37-8.33 (m, 2H), 7.99 (d, J=8.1 Hz, 1H), 7.94 (d, J=8.1 Hz, 1H), 7.79-7.74 (m, 3H), 7.70 (t, J=7.4 Hz, 1H), 7.67-7.64 (m, 2H), 6.09 (q, J=6.3 Hz, 1H), 5.75 (d, J=6.2 Hz, 1H), 3.97-3.89 (m, 2H); ^{13}C NMR (125.7 MHz, DMSO−d_6) δ [ppm]: 161.93, 160.03, 142.13, 141.47, 136.70, 136.20, 130.64, 130.58, 127.86, 127.84, 127.82, 127.76, 127.64, 126.31, 126.01, 120.94, 119.68, 72.11, 41.72.

Synthesis of (E)-1,2-di(isoquinolin-1-yl)ethene (**46**)

A stirred suspension of **45** (1.700 g, 5.67 mmol) in Ac_2O (15 ml) was heated to 70 °C with a water bath. A clear solution was obtained after 10 min and a solid started to precipitate after 20 min. After 5 h, the mixture was poured in 150 ml of water and $NaHCO_3$ was added under stirring

till pH 8. The mixture was stirred overnight, 200 ml of water was added, and the light brown solid was filtered washing with 30 ml of distilled water, EtOH (10 ml) and Et_2O (30 ml). The solid was collected and dried in a vacuum at 50 ◦ C (1.395 g, 4.94 mmol, yield 87%, m.p.: 260 ◦ C (dec.)).

^{1}H NMR (500 MHz, CDCl) δ [ppm]: 9.03 (s, 2H), 8.67-8.65 (m, 4H), 7.88 (d, J=7.9 Hz, 2H), 7.76-7.69 (m, 6H). ^{13}C NMR (125.7 MHz, CDCl 3) δ [ppm]: 153.94, 142.23, 136.99, 130.42, 129.59, 127.73, 127.48, 127.35, 125.16, 121.10.

Synthesis of (E)-1,1'-(ethene-1,2-diyl)bis(2-methylisoquinolinium) trifluoromethanesulfonate (**5**)

Under N_2 atmosphere, MeOTf (95 mg, 0.58 mmol) was added to an ice-cooled stirred suspension of 46 (50 mg, 0.18 mmol) in dry $MeNO_2$ (2.0 ml). The mixture was kept under stirring at 0 ◦ C for 6 h obtaining a clear solution. Product was precipitated as a white solid after addition of Et 2 O (6 ml), collected by filtration on an Hirsh funnel, and dried in a vacuum (45 mg, 0.073 mmol, yield 42%).

^{1}H NMR (500 MHz, CD_3CN) δ [ppm]: 8.78 (d, J=8.8 Hz, 2H), 8.55 (d, J=6.9 Hz, 2H), 8.50 (d, 6.8 Hz, 2H), 8.40 (d, 8.3 Hz, 2H), 8.33 (t, J=7.7 Hz, 2H), 8.16 (t, J=7.8 Hz, 2H), 7.80 (s, 2H), 4.42 (s, 6H); ^{13}C NMR (125.7 MHz, CD_3CN) δ [ppm]: 154.19, 138.38, 136.98, 136.78, 133.46, 132.32, 129.19, 128.33, 127.11, 126.35, 47.52.

DERIVATIVE 9 **FiQ-Q**

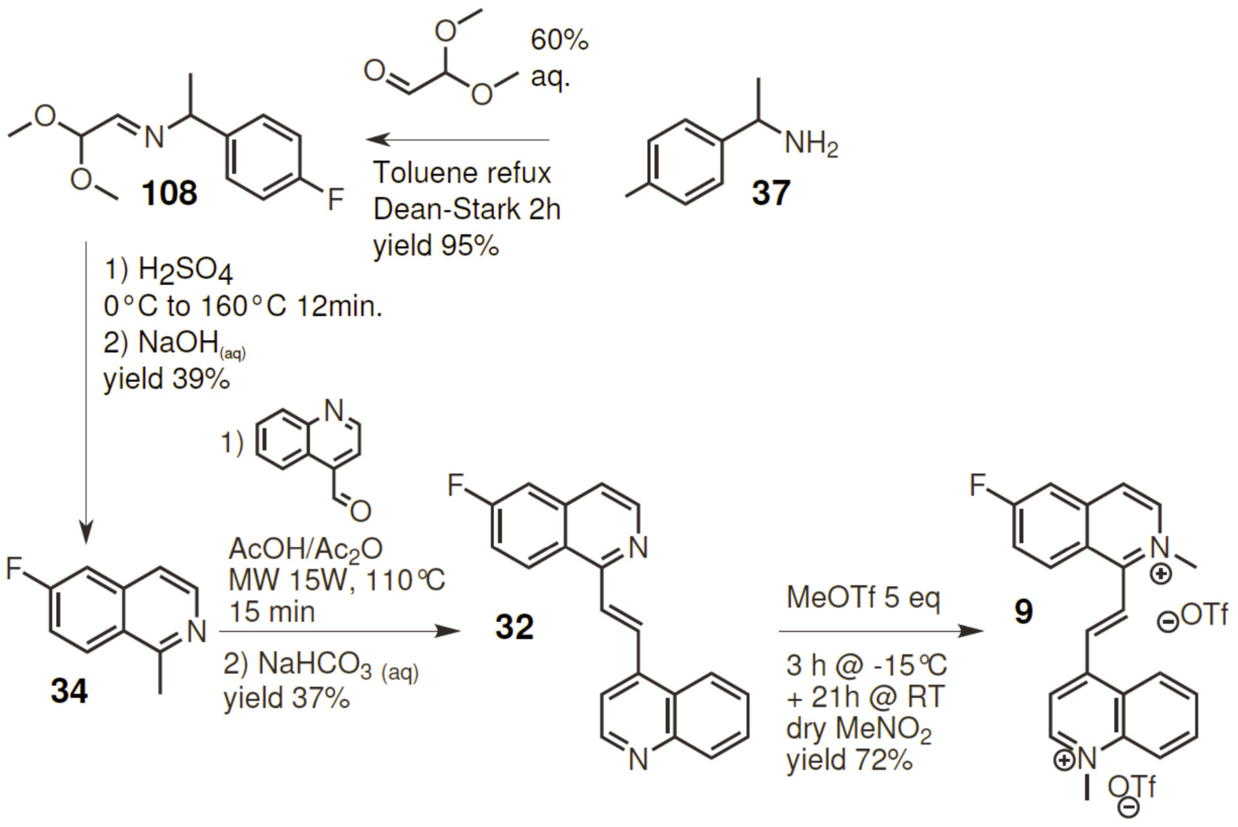

Synthesis of (E)-N-(2,2-dimethoxyethylidene)-1-(4-fluorophenyl)-ethanamine (**108**)

A mixture of 1-(4-fluorophenyl)ethanamine (**37**) (5.000 g, 35.92 mmol) and dimethoxyacetaldehyde 60% w/w aqueous solution (6.856 g, 39.52 mmol) in 35 ml of toluene were refluxed for 2 h under nitrogen atmosphere, collecting water with a Dean-Stark apparatus. The mixture was cooled to room temperature and solvent was removed under reduced pressure (□1 mmHg) obtaining product as a yellow oil (7.66 g, 34.0 mmol, yield 95%).

^{1}H NMR (500 MHz, DMSO−d_6) δ [ppm]: 7.63 (d, J=5.0 Hz, 1H), 7.39-7.36 (m, 2H), 7.17-7.14 (m, 2H), 4.65 (d, J=5.0 Hz, 1H), 4.45 (q, J=6.6 Hz, 1H), 3.33 (s, 3H), 3.28 (s, 3H), 1.39 (d, J=6.6 Hz, 3H).

Synthesis of 6-fluoro-1-methylisoquinoline (**34**)

In a 500 ml RBF, 108 (7.600 g, 33.76 mmol) was slowly added, under stirring, to ice cooled H_2SO_4 (76 ml). The solution was heated to 160°C in 10 min and kept at that temperature for 2 min. The mixture was slowly poured in ice (400 g) and basified till pH 9 adding NaOH. The mixture was steam distilled till a clear distillate was obtained. The distillate was extracted with 4 × 200ml of CH_2Cl_2. The organic phase was collected, dried over Na_2SO_4 and evaporated under reduced pressure obtaining product as a colourless liquid (2.095 g, 13.00 mmol, yield 39%).

^{1}H NMR (500 MHz, $CDCl_3$) δ [ppm]: 8.37 (d, J=5.8 Hz, 1H), 8.12 (dd, J=9.2 Hz, J=5.5 Hz, 1H), 7.45 (d, J=5.8 Hz, 1H), 7.39 (dd, J=9.3 Hz, J=2.5 Hz, 1H), 7.33 (td, J=8.7 Hz, J=2.5 Hz, 1H), 2.94 (s, 3H); ^{13}C NMR (125.7 MHz, $CDCl_3$) δ [ppm]: 163.03 (d, J=252.0 Hz), 158.64, 142.92, 137.67 (d, J=10.2 Hz), 128.88 (d, J=9.6 Hz), 124.82, 119.10 (d, J=4.9 Hz), 117.39 (d, J=25.0 Hz), 110.52 (d, J=20.5 Hz), 22.64.

Synthesis of (E)-4-(2-(6-fluoroisoquinolin-1-yl)vinyl)quinoline (**32**)

A suspension of 34 (200 mg, 1.24 mmol) and quinoline-4-carboxyaldehyde (163 mg, 1.04 mmol) in a mixture of AcOH (0.5 ml) and Ac_2O (0.2 ml) was heated in a CEM discover microwave reactor for 15 min (P max =15 W, 110 °C. The solution was allowed to cool to RT observing the crystallization of a yellow solid which was recovered and dried on filter paper. Product was purified by crystallization from acetone/AcOH 20:1. The crystalline solid was suspended in 25 ml of a saturated $NaHCO_3$ aqueous solution for 30 min and extracted with 2 × 20ml of CH_2Cl_2. The organic phase was collected, dried over Na_2SO_4 and evaporated under reduced pressure at 80 °C obtaining product as a white solid (138 mg, 0.459 mmol, yield 37%, m.p.: 170-171 °C).

^{1}H NMR (500 MHz, $CDCl_3$) δ [ppm]: 8.98 (d, J=4.6 Hz, 1H), 8.76 (d, J=15.3 Hz, 1H), 8.64 (d, J=5.6 Hz, 1H), 8.42 (dd, J=9.3 Hz, J=5.3 Hz, 1H), 8.38 (d, J=8.3 Hz, 1H), 8.22 (d, J=8.4 Hz, 1H), 8.16 (d, J=15.3 Hz, 1H), 7.81-7.77 (m, 2H), 7.66-7.63 (m, 2H), 7.49 (dd, J=9.1 Hz, J=2.5 Hz, 1H), 7.44 (td, J=8.7 Hz, J=2.6 Hz, 1H). Anal. Calcd for $C_{20}H_{13}FN_2$: C, 79.98; H, 4.36; N, 9.33. Found: C, 80.06; H, 5.87; N, 9.22.

Synthesis of (E)-4-(2-(6-fluoro-2-methylisoquinolinium-1-yl)vinyl)-1-methylquinolinium trifluoromethanesulfonate (**9**)

Under N_2 atmosphere, MeOTf (222 mg, 1.35 mmol) was added to a stirred suspension of 32 (80 mg, 0.266 mmol) in dry $MeNO_2$ (2 ml) cooled to −15 °C with an ice/salt bath. The mixture was kept under stirring at −15 °C for 3 h obtaining a clear solution, and stirred for 21 h at room temperature. Product was precipitated as a pale yellow solid after addition of Et_2O (10 ml), collected by filtration and dried in a vacuum (120 mg, 0.191 mmol, yield 72%, m.p.: 224.4-225.5 ◦ C).

^{1}H NMR (500 MHz, DMSO−d_6) δ [ppm]: 9.68 (d, J=6.2 Hz, 1H), 8.91 (d, J=6.9 Hz, 1H), 8.81 (dd, J=9.4 Hz, J=5.2 Hz, 1H), 8.77 (d, J=6.2 Hz, 1H), 8.68 (d, J=8.5 Hz, 1H), 8.62-8.59 (m, 2H), 8.44 (d, J=16.3 Hz, 1H), 8.38-8.32 (m, 2H), 8.29 (dd, J=9.1 Hz, J=2.5 Hz, 1H), 8.12 (t, J=7.7 Hz, 1H), 8.03 (td, J=9.2 Hz, J=2.1 Hz, 1H), 4.72 (s, 3H), 4.47 (s, 3H); ^{13}C NMR (125.7 MHz, DMSO−d_6) δ [ppm]: 166.44 (d, J=260.0 Hz), 156.17, 150.233, 150.09, 140.51 (d, J=12.9 Hz), 139.27, 138.41, 137.64, 136.05, 134.74 (d, J=11.0 Hz), 130.85, 129.59, 127.47, 126.94 , 125.21 (d, J=4.9 Hz), 124.97, 122.45 (d, J=25.6 Hz), 120.40, 119.96, 112.37 (d, J=22.4 Hz), 47.50, 46.17; Anal. Calcd for $C_{24}H_{19}F_7N_2O_6S_2$: C, 45.86; H, 3.05; N, 4.46; S, 10.2. Found: C, 46.63; H, 3.41; N, 4.66; S, 11.23.

DERIVATIVE 10, **iQ-ClQ**

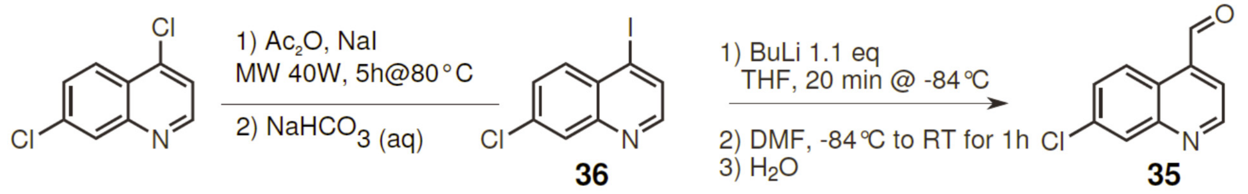

Synthesis of 7-chloro-4-iodoquinoline (**36**)

In a 35 ml microwave reaction vessel, a mixture of 4,7-dichloroquinoline (4.500 g, 22.72 mmol) and NaI (10.217 g, 68.16 mmol) in Ac_2O (5.798 g, 56.8 mmol) was prepared and heated at 80 °C for 5 h in a CEM Discover microwave reactor (P max =40 W). Product was suspended in 100 ml of $NaHCO_3$ (aq) 5% and stirred for 1 h. A 5% aqueous solution of Na_2SO_3 was added (50 ml) followed by a 5% aqueous solution of $Na_2S_2O_3$ (50 ml). The mixture was extracted with 3 × 30ml of CH_2Cl_2, and dried over Na_2SO_4. Solvent was evaporated under reduced pressure obtaining product as a white solid which was further purified by crystallization from EtOH (5.011 g, 17.31 mmol, yield 76%, m.p.: 123-124 °C).

^1H NMR (500 MHz, $CDCl_3$) δ [ppm]: 8.44 (d, J=4.5 Hz, 1H), 8.06 (d, J=2.1 Hz, 1H), 7.99 (d, J=4.6 Hz, 1H), 7.97 (d,J=9.0 Hz, 1H), 7.57 (dd, J=8.9 Hz, J=2.1 Hz, 1H).

Synthesis of 7-chloroquinoline-4-carbaldehyde (**35**)

Under N_2 atmosphere, a solution of **36** (5.000 g, 17.27 mmol) in anhydrous THF (30 ml) was prepared, and cooled to −84 °C with an AcOEt/N_2 slush bath. A 1.28 M BuLi solution in hexane (14.83 ml) was slowly added under stirring. The obtained red-brown mixture was stirred at −84 °C for 20 min. Anhydrous DMF (1.262 g, 17.27 mmol) was slowly added and the mixture

was allowed to warm slowly to room temperature and kept under stirring for 1 h. Water (50 ml) was added to the mixture obtaining an orange solution which was extracted with 3 × 50ml of AcOEt. The organic phase was collected, washed with brine (100 ml), dried over Na_2SO_4 and evaporated under reduced pressure. The obtained product was purified by column chromatography (SiO_2, eluent: AcOEt/n−Hex 1:3) (1.136 g, 5.93 mmol, yield 34 %, m.p.: 105-106 ◦ C).

^{1}H NMR (500 MHz, $CDCl_3$) δ [ppm]: 10.45 (s, 1H), 9.21 (d, J=4.2 Hz, 1H), 9.02 (d, J=9.1 Hz, 1H), 8.22 (d, J=2.2 Hz, 1H), 7.80 (d, J=4.2 Hz, 1H), 7.69 (dd, J=9.1 Hz, J=2.2 Hz, 1H).

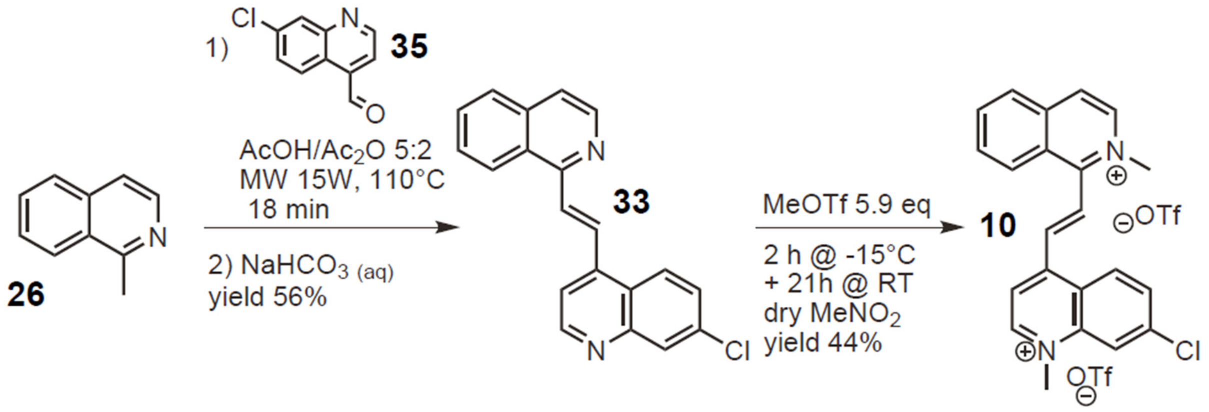

Synthesis of (E)-7-chloro-4-(2-(isoquinolin-1-yl)vinyl)quinoline (**33**)

A suspension of **26** (374 mg, 2.61 mmol) and **35** (500 mg, 2.61 mmol) in 2 ml of a 5:2 AcOH/Ac_2O mixture was heated in a CEM discover microwave reactor for 18 min (P max =15 W, 110 °C). The solution was allowed to cool to RT observing the crystallization of a yellow solid which was suspended in 30 ml of water and filtered on a Hirsh funnel. Product was purified by crystallization from acetone/AcOH 19:1. The crystalline solid was suspended in 100 ml of saturated $NaHCO_3$ aqueous solution for 30 min and extracted with 3 × 50ml of CH_2Cl_2 . The organic phase was collected, dried over Na_2SO_4 and evaporated under reduced pressure

obtaining product as a white solid. Product was kept for 1 day in a desiccator to remove residual water (467 mg, 1.474 mmol, yield 56%, m.p.: 163-164 ◦ C).

^{1}H NMR (500 MHz, $CDCl_3$) δ [ppm]: 8.97 (d, J=4.6 Hz, 1H), 8.71 (d, J=15.4 Hz, 1H), 8.65 (d, J=5.6 Hz, 1H), 8.38 (d, J=8.5 Hz, 1H), 8.33 (d, J=9.0 Hz, 1H), 8.22 (d, J=15.3 Hz, 1H), 8.17 (d, J=2.1 Hz, 1H), 7.90 (d, J=8.2 Hz, 1H), 7.76-7.73 (m, 2H), 7.70-7.67 (m, 2H), 7.57 (dd, J=9.0 Hz, J=2.1 Hz, 1H).

Synthesis of (E)-7-chloro-1-methyl-4-(2-(2-methylisoquinolinium-1-yl)vinyl)quinolinium trifluoromethanesulfonate (**10**)

Under N_2 atmosphere, MeOTf (310 mg, 1.89 mmol) was added to a stirred suspension of **33** (100 mg, 0.316 mmol) in dry $MeNO_2$ (2 ml) cooled to −15 °C with an ice/salt bath. The mixture was kept under stirring at −15 °C for 2 h obtaining a clear solution, and stirred for 21 h at room temperature. Product was precipitated after addition of Et_2O (5 ml) and acetone (1 ml), collected by filtration on a Hirsh funnel, and dried in a vacuum. Product was purified by crystallization from EtOH (89 mg, 0.138 mmol, yield 44%, m.p.: 159.5-159.9 ◦ C).

^{1}H NMR (500 MHz, DMSO−d_6) δ [ppm]: 9.68 (d, J=6.3 Hz, 1H), 8.91 (d, J=6.8 Hz, 1H), 8.79-8.78 (m, 2H), 8.71 (d, J=9.2 Hz, 1H), 8.67-8.66 (m, 2H), 8.45-8.41 (m, 2H), 8.36 (d, J=16.3 Hz, 1H), 8.32 (t, J=7.7 Hz, 1H), 8.20 (dd, J=9.2 Hz, J=1.9 Hz, 1H), 8.09 (t, J=7.3 Hz, 1H), 4.70 (s, 3H), 4.49 (s, 3H); Anal. Calcd for $C_{24}H_{19}F_6N_2O_6S_2Cl$: C, 44.69; H, 2.97; N, 4.34. Found: C, 44.89; H, 3.06; N, 4.41.

DERIVATIVE 11, **twQ-iQ**

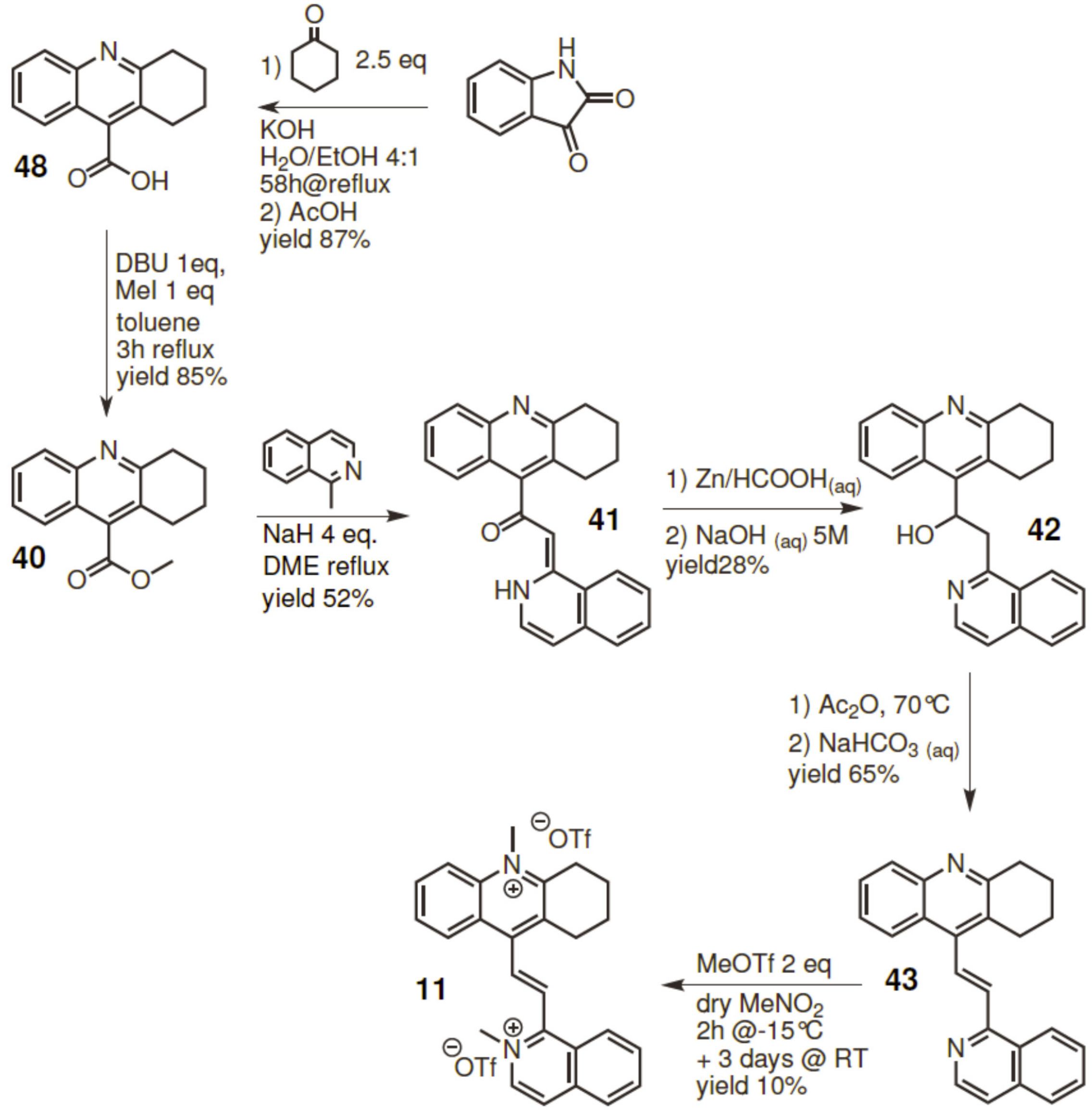

Synthesis of 1,2,3,4-tetrahydroacridine-9-carboxylic acid (**48**)

A solution of KOH (63.0 g, 1.12 mol) in EtOH/H_2O 1:4 (200 ml) was slowly added under stirring to a mixture of isatin (50.00 g, 339.8 mmol) and cyclohexanone (84.00 g, 849.6 mmol). The obtained deep red solution was heated to reflux observing a colour change to pale yellow

after 30 min with the formation of a biphasic mixture. After refluxing for 58 h, water (200 ml) was added followed by AcOH till pH 4 observing the formation of a white precipitate. The mixture was stirred for 20 min, and the filtrate was collected by filtration washing with water 200 ml, MeOH (200 ml) and Et_2O (50 ml). Residual solvent was evaporated under reduced pressure at 60 ◦ C for 2 days and the solid was further dried in a desiccator over P_2O_5 for 3 days (64.13 g, 295.2 mmol, yield 87%, m.p.: >300 ◦ C)

^{1}H NMR (500 MHz, DMSO−d_6) δ [ppm]: 7.93 (d, J=8.4 Hz, 1H), 7.72-7.69 (m, 2H), 7.57 (t, J=7.6 Hz, 1H), 3.18 (s, 2H), 3.06 (t, J=6.6 Hz, 2H), 2.90 (t, J=6.4 Hz, 2H), 1.94-1.89 (m, 2H), 1.87-1.82 (m, 2H).

Synthesis of methyl 1,2,3,4-tetrahydroacridine-9-carboxylate (**40**)

To a stirred suspension of **48** (10.00 g, 44.00mmol) and DBU (6.698 g, 44.00 mmol) in toluene (75 ml), MeI (6.245 g, 44.00 mmol) was added; the mixture was stirred at RT for 20 min and then heated to reflux for 3 h. The change in solution colour to yellow and the formation of a white precipitate were observed. The mixture was cooled to RT and filtered washing with 30 ml of toluene. The filtrate was collected, and solvent was evaporated under reduced pressure obtaining product as a pale yellow solid (8.988 g, 37.25 mmol, yield 85%, m.p.: 68-70 °C).

^{1}H NMR (500 MHz, $CDCl_3$) δ [ppm]: 8.05 (d, J=8.5 Hz, 1H), 7.67-7.64 (m, 2H), 7.49 (t, J=7.5 Hz, 1H), 4.05 (s, 3H), 3.18 (t, J=6.6 Hz, 2H), 2.93 (t, J=6.4 Hz, 2H), 2.01-1.96 (m, 2H), 1.92-1.88 (m, 2H).

Synthesis of (Z)-2-(isoquinolin-1(2H)-ylidene)-1-(1,2,3,4-tetrahydroacridin-9-yl)ethanone (**41**)

Under N_2 atmosphere, in an oven dried 250 ml 3-necked RBF, fitted with reflux condenser, septum and dropping funnel, a suspension of NaH (828 g, 20.72 mmol) in anhydrous DME (10 ml) was prepared. A solution of freshly distilled 26 (742 mg, 5.18 mmol) and 40 (1.500 g, 6.22 mmol) in 20 ml of anhydrous DME was drop wise added to the refluxing suspension of NaH. The suspension was refluxed for a total of 46 h observing the formation of a bright yellow solution. The mixture was cooled with an ice-water bath, and AcOH (2.50 g) was added followed by 20 ml of water. A yellow solid precipitated and was filtered washing with H 2 O (50 ml). Product was dried in a desiccator for 3 days and crystallized from DMF (950 mg, 2.70 mmol, yield 52%, m.p.: 240 °C (dec)).

^{1}H NMR (500 MHz, $CDCl_3$) δ [ppm]: 15.96 (s, 1H), 8.09 (d, J=6.8 Hz, 1H); 8.03 (d, J=8.4 Hz, 1H), 7.94 (d, J=8.3 Hz, 1H), 7.71 (t, J=7.1 Hz, 1H), 7.66-7.62 (m, 2H), 7.51-7.48 (m, 2H), 7.42 (t, J=7.4 Hz, 1H), 6.97 (d, J=6.8 Hz, 1H), 6.20 (s, 1H), 3.24 (t, J=6.3 Hz, 2H), 3.16-2.94 (m, 2H), 2.00 (qui, J=6.0 Hz, 2H), 1.96-1.79 (m, 2H).

Synthesis of 2-(isoquinolin-1-yl)-1-(1,2,3,4-tetrahydroacridin-9-yl)-ethanol (**42**)

To a stirred mixture of 41 (900 mg, 2.553 mmol) and Zn (501 mg, 7.661 mmol), aqueous formic acid 50% (50 ml) was added. The solution rapidly changed its colour from orange to pale green. The mixture was kept under stirring for 20 min and filtered collecting the filtrate in an ice-cooled stirred 5 M NaOH solution (150 ml). A white solid separated, and the mixture was kept under stirring for 15 min. The suspension was filtered and washed with 50 ml of 5 M NaOH solution followed by 100 ml of deionized water. The white solid was dried in a desiccator for 2 days (256 mg, 0.722 mmol, yield 28%, m.p.: 80 °C (dec.)).

^{1}H NMR (500 MHz, $CDCl_3$) δ [ppm]: 8.90 (broad s, 1H), 8.51 (d, J=5.8 Hz, 1H), 8.02 (d, J=8.3 Hz, 1H), 7.96 (d, J=8.5 Hz, 1H), 7.87 (d, J=8.2 Hz, 1H), 7.71 (t, J=7.6 Hz, 1H), 7.65 (d, J=5.8 Hz, 1H), 7.62 (td, J=8.3 Hz, J=1.1 Hz, 1H), 7.56 (t, J=7.7 Hz, 1H), 7.44 (td, J=7.1 Hz, 1.4 Hz, 1H), 6.45 (d, J=10.1 Hz, 1H), 4.13-4.08 (m, 1H), 3.57 (dd, J=17.4 Hz, J=2.0 Hz, 1H), 3.23 (t, J=6.4 Hz, 1H), 3.17 (t,J=6.4 Hz, 2H), 1.97-1.85 (m, 6H).

Synthesis of (E)-9-(2-(isoquinolin-1-yl)vinyl)-1,2,3,4-tetrahydroacridine (**43**)

A suspension of **42** (200 mg, 0.564 mmol) in Ac_2O (2 ml) was heated to 60 °C in a CEM Discover Microwave reactor (P max =15 W, cooling off) for 3 h. The formation of a white precipitate was observed. The mixture was poured in a saturated $NaHCO_3$ solution, stirred for 20 min, and extracted with 3 × 25ml of CH_2Cl_2. The organic phase was collected, washed with brine, dried over Na_2SO_4 and evaporated under reduced pressure. The obtained brown solid was sonicated with few ml of i-PrOH and filtered washing with few ml of Et_2O. Residual solvent was evaporated under reduced pressure at 50 °C (123 mg, 0.366 mmol, yield 65%. m.p.: 123.4-124.6 °C).

^{1}H NMR (500 MHz, $CDCl_3$) δ [ppm]: 8.65 (d, J=5.6 Hz, 1H), 8.27-8.21 (m, 3H), 7.89 (d, J=8.2 Hz, 1H), 7.78 (d, J=15.9 Hz, 1H), 7.73-7.67 (m, 3H), 7.62 (t, J=7.6 Hz, 1H), 7.49 (t, 7.4 Hz, 1H), 3.27 (broad s, 2H), 3.07 (t, J=6.3 Hz, 2H), 2.04-1.97 (m, 2H), 1.95-1.90 (m, 2H).

Synthesis of (E)-10-methyl-9-(2-(2-methylisoquinolinium-1-yl)-vinyl)-1,2,3,4-tetrahydroacridinium trifluoromethanesulfonate (**11**)

Under N_2 atmosphere, MeOTf (97 mg, 0.59 mmol) was added to an ice-cooled stirred suspension of 43 (100 mg, 0.297 mmol) in dry $MeNO_2$ (0.5 ml). The mixture was kept under stirring at −15 °C for 2 h obtaining a clear solution. The mixture was allowed to warm to RT and kept under stirring for 3 days. Product was precipitated as a pale brown solid after addition of Et_2O (10 ml), collected by filtration and purified by crystallization from i-PrOH (20 mg, 0.030 mmol, yield 10%, m.p.: 155.2-156.7 °C)

^{1}H NMR (500 MHz, CD_3CN) δ [ppm]: 8.79 (d, J=8.6 Hz, 1H), 8.56 (d, J=8.4 Hz, 1H), 8.52-8.48 (m, 2H), 8.44 (d, J=6.7 Hz, 1H), 8.37 (d, J=8.2 Hz, 1H), 8.30 (t, J=7.9 Hz, 1H), 8.23 (t, J=8.1 Hz, 1H), 8.14 (t, J=7.6 Hz, 1H), 8.02 (t, J=7.6 Hz, 1H), 7.76 (d, J=17.0 Hz, 1H), 7.49 (d, J=17.0 Hz, 1H), 4.48 (s, 3H), 4.40 (s, 3H), 3.45 (t, J=6.3 Hz, 2H), 3.20 (t, J=6.3 Hz, 2H), 2.14-2.08 (m, 4H); 13 C NMR (125.7 MHz, CD_3CN) δ [ppm]: 160.74, 155.66, 149.74, 138.96, 138.90, 138.28, 136.80, 136.58, 134.73, 132.37, 132.04, 129.69, 129.51, 128.79, 128.22, 127.50, 127.30, 126.04, 125.76, 119.07, 47.38, 39.36, 31.17, 28.30, 21.10, 20.51; Anal. Calcd for $C_{28}H_{26}F_6N_2O_6S_2$: C, 50.60; H, 3.94; N, 4.21. Found: C, 51.05; H, 3.77; N, 4.20.

2. Electrochemistry of Q-Q derivate

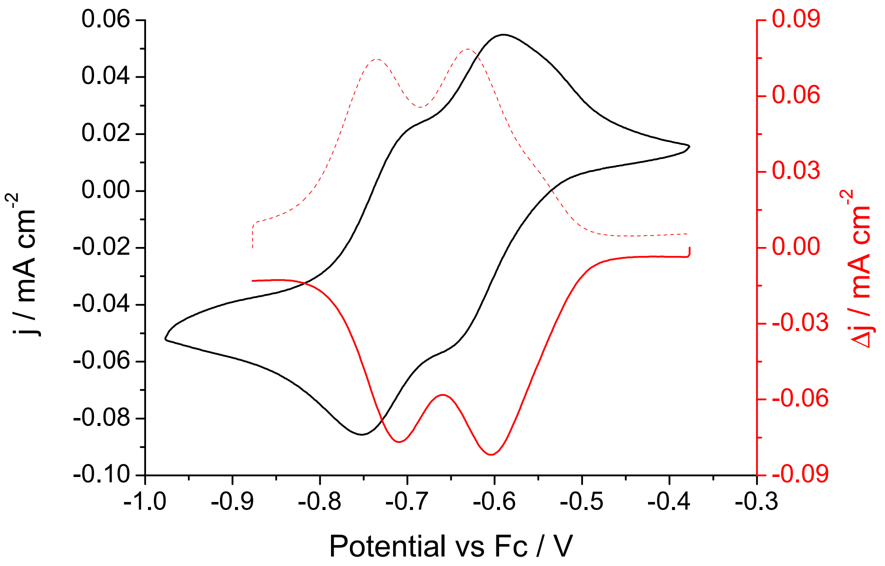

Figure SI_1: Comparison between the coefficient of variation (CV) (black) and the DPV (red) for the **Q-Q** compound. The DPV analysis shows a consistent peak to peak separation of 100 mV. The DPV has been obtained with the same apparatus using a scan rate of 20 mV/s.

Table SI_1: Synthesized compounds and codes referred to the synthetic routes. Data obtained at the B3LYP/6-31G(d) level of the theory. The reported C-C distances are averaged values.

Code	Name	Dihedral angle between quinolinic rings (°)			C-C Distance (Å)			
					central	C-ring	central	C-ring
		2+	1+	neu	2+	2+	neu	neu
10	iQ-ClQ	57.0	31.2	26.8	1.3477	1.4727	1.4358	1.3760
9	FiQ-Q	89.1	31.0	17.6	1.3475	1.4812	1.4340	1.3780
5	iQ-iQ	79.4	30.2	18.2	1.3466	1.4810	1.4400	1.3775
4	iQ-Q	89.3	30.8	0	1.3476	1.4739	1.4332	1.3835
7	Q-Q	0	25.3	8.2	1.3557	1.4674	1.4346	1.3793
	Q-(CN)P	0	0	0	1.3673	1.4797	1.4502	1.3860
11	twQ-iQ	72.5	58.3	29.5	1.3470	1.4792	1.4367	1.3724
